# Polygenic risk scores associate with blood pressure traits across the lifespan

**DOI:** 10.1093/eurjpc/zwad365

**Published:** 2023-11-26

**Authors:** Karsten Øvretveit, Emma M L Ingeström, Michail Spitieris, Vinicius Tragante, Kaitlin H Wade, Laurent F Thomas, Brooke N Wolford, Ulrik Wisløff, Daniel F Gudbjartsson, Hilma Holm, Kari Stefansson, Ben M Brumpton, Kristian Hveem

**Affiliations:** K.G. Jebsen Centre for Genetic Epidemiology, Faculty of Medicine and Health Sciences, Department of Public Health and Nursing, Norwegian University of Science and Technology (NTNU), Postboks 8905, N-7491 Trondheim, Norway; Department of Circulation and Medical Imaging, Norwegian University of Science and Technology (NTNU), Trondheim, Norway; K.G. Jebsen Centre for Genetic Epidemiology, Faculty of Medicine and Health Sciences, Department of Public Health and Nursing, Norwegian University of Science and Technology (NTNU), Postboks 8905, N-7491 Trondheim, Norway; Department of Mathematical Sciences, Norwegian University of Science and Technology (NTNU), Trondheim, Norway; deCODE genetics/Amgen Inc., Reykjavik, Iceland; MRC Integrative Epidemiology Unit, University of Bristol, Bristol BS8 1TH, UK; Population Health Science, Bristol Medical School, Bristol BS8 1TH, UK; Avon Longitudinal Study of Parents and Children, Bristol BS8 1TH, UK; K.G. Jebsen Centre for Genetic Epidemiology, Faculty of Medicine and Health Sciences, Department of Public Health and Nursing, Norwegian University of Science and Technology (NTNU), Postboks 8905, N-7491 Trondheim, Norway; Department of Clinical and Molecular Medicine, Norwegian University of Science and Technology (NTNU), Trondheim, Norway; K.G. Jebsen Centre for Genetic Epidemiology, Faculty of Medicine and Health Sciences, Department of Public Health and Nursing, Norwegian University of Science and Technology (NTNU), Postboks 8905, N-7491 Trondheim, Norway; Department of Circulation and Medical Imaging, Norwegian University of Science and Technology (NTNU), Trondheim, Norway; deCODE genetics/Amgen Inc., Reykjavik, Iceland; School of Engineering and Natural Sciences, University of Iceland, Reykjavik, Iceland; deCODE genetics/Amgen Inc., Reykjavik, Iceland; deCODE genetics/Amgen Inc., Reykjavik, Iceland; Faculty of Medicine, University of Iceland, Reykjavik, Iceland; K.G. Jebsen Centre for Genetic Epidemiology, Faculty of Medicine and Health Sciences, Department of Public Health and Nursing, Norwegian University of Science and Technology (NTNU), Postboks 8905, N-7491 Trondheim, Norway; HUNT Research Centre, Department of Public Health and Nursing, Norwegian University of Science and Technology, Levanger, Norway; K.G. Jebsen Centre for Genetic Epidemiology, Faculty of Medicine and Health Sciences, Department of Public Health and Nursing, Norwegian University of Science and Technology (NTNU), Postboks 8905, N-7491 Trondheim, Norway; Department of Innovation and Research, St. Olavs Hospital, Trondheim, Norway

**Keywords:** HUNT, deCODE, ALSPAC, Polygenic risk scores, Blood pressure, Hypertension

## Abstract

**Aims:**

Hypertension is a major modifiable cause of morbidity and mortality that affects over 1 billion people worldwide. Blood pressure (BP) traits have a strong genetic component that can be quantified with polygenic risk scores (PRSs). To date, the performance of BP PRSs has mainly been assessed in adults, and less is known about polygenic hypertension risk in childhood.

**Methods and results:**

Multiple PRSs for systolic BP (SBP), diastolic BP (DBP), and pulse pressure were developed using either genome-wide significant weights, pruning and thresholding, or Bayesian regression. Among 87 total PRSs, the top performer for each trait was applied in independent cohorts of children and adult to assess genotype-phenotype associations and disease risk across the lifespan. Differences between those with low (1st decile), average (2nd–9th decile), and high (10th decile) PRS emerge in the first years of life and are maintained throughout adulthood. These diverging BP trajectories also seem to affect cardiovascular and renal disease risk, with increased risk observed among those in the top decile and reduced risk among those in the bottom decile of the polygenic risk distribution compared with the rest of the population.

**Conclusion:**

Genetic risk factors are associated with BP traits across the lifespan, beginning in the first years of life. Given the importance of exposure time in disease pathogenesis and the early rise in BP levels among those genetically susceptible, PRSs may help identify high-risk individuals prior to hypertension onset, facilitate primordial prevention, and reduce the burden of this public health challenge.

## Graphical Abstract

**Graphical Abstract:**
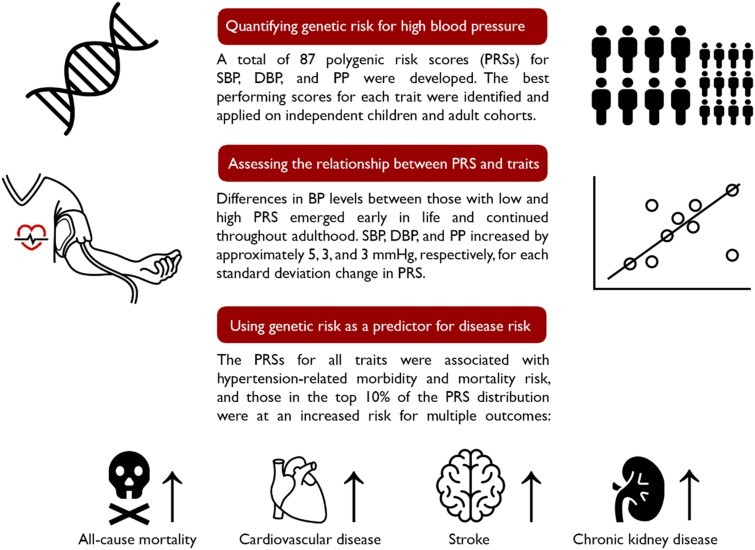

## Introduction

Hypertension is a leading, modifiable cause of morbidity and mortality worldwide.^1,2^ It can be broadly defined as a persistently elevated arterial blood pressure (BP) at or above a level at ‘which investigation and treatment do more good than harm’.^3^ The threshold for pharmacological intervention varies between treatment guidelines but is generally indicated at a systolic BP (SBP) ≥ 140 mmHg and/or a diastolic BP (DBP) ≥ 90 mmHg.^4,5^ However, the risk of cardiovascular, cerebrovascular, and renal disease may increase at a BP below what is considered normal,^6^ with 30% of the SBP-related burden occurring in the 115–140 mmHg range.^7^ Most hypertension cases can be described as primary, or essential, with no known underlying cause. Although prevalence has decreased slightly in high-income countries, with improvements in BP awareness, treatment, and control, the opposite is true for low- and middle-income countries, where prevalence has increased with little change in prevention and management.^8^

The multifactorial aetiology of essential hypertension makes prevention and management challenging.^4,5^ There is substantial interindividual and between-population variation in BP, with population-specific heritability ranging from 6 to 68%.^9^ The estimates also vary based on measurement procedures, such as single, average, or long-term measurements.^10^ Genome-wide association studies (GWAS) with robust sample sizes have identified over 1000 sequence variants associated with BP traits,^11–15^ but the variance explained is still in single digit percentages. Despite the remaining missing heritability for these and other common phenotypes, recent genomic and computational advances have substantially improved the predictive power of genetic prediction using polygenic risk scores (PRSs) for a range of diseases, approaching the risk conveyed by monogenic mutations.^16^

A major clinical potential of PRSs is identifying high-risk individuals that are likely to benefit from preventive measures. Recent studies indicate that genome-wide PRSs for BP traits can improve clinical risk prediction of hypertension in adults.^17,18^ Moreover, the contrasting BP levels in both tails of the polygenic risk spectrum appear to translate into appreciable differences in cardiovascular and renal disease risk. Yet, the point in the lifespan at which phenotypic expression starts diverging as a function of a genetic risk remains unclear. Given that the duration of exposure to high BP levels is a key driver of disease risk, application of PRSs in individuals at all stages of life is warranted. To this end, we derive, train, and apply multiple PRSs for BP traits in large cohorts of children and adults, and leverage the longitudinal nature and repeated BP measurements in these cohorts to investigate the association between PRSs, BP traits, and disease risk, from the first to the last years of life.

## Methods

### Study cohorts

We included a total of 174 493 genotyped and phenotyped individuals of European ancestry from the Trøndelag Health Study (HUNT; *n* = 86 569), deCODE genetics (*n* = 81 117), and the Avon Longitudinal Study of Parents and Children (ALSPAC) study (*n* = 6 807). The HUNT study is Norway’s largest on-going population-based study and has enrolled ∼240 000 individuals to date, of which more than 100 000 have contributed biological material.^22,23^ Data collection began with HUNT1 (1984–86) and has been repeated approximately every decade since, with HUNT4 being completed in 2019. The deCODE genetics hypertension study uses BP measures and information on cardiovascular diagnoses from Iceland. Here, we leverage genotype data derived from whole-genome sequencing of 28 075 Icelanders^24^ and imputed into 81 117 chip-genotyped persons.^25^ In total, up to 31.7 million variants have been characterized in this population. The ALSPAC study is a multi-generational prospective birth cohort study that recruited pregnant women with expected dates of delivery 1 April 1991 to 31 December 1992 in Avon, UK, between 1991 and 1992 and followed them and their partners and offspring for over two decades.^26–28^ The initial number of pregnancies enrolled was 14 541, and of these, there was a total of 14 676 foetuses, resulting in 14 062 live births and 13 988 children who were alive at 1 year of age. Since then, these children have been followed up regularly with questionnaires and clinical measurements until the present day. An additional 913 have been enrolled beginning when the oldest children were ∼7 years old, bringing the total sample size for analyses using data collected after this age to 15 454 pregnancies that resulted in 15 589 foetuses, 14 901 of which were alive at 1 year of age. This study included 6807 children with genotype and phenotype data from birth until 24 years of age. Study data were collected and managed using REDCap electronic data capture tools hosted at the University of Bristol.^29^ REDCap (Research Electronic Data Capture) is a secure, web-based software platform designed to support data capture for research studies. Detailed information on genotyping procedures for each sample is available in the Supplementary material online.

### Polygenic risk scores

A PRS provides an estimate of inherited risk of a given polygenic trait based on association coefficients between genetic markers and the phenotype of interest. To determine the genetic susceptibility to elevated BP, we compared four different methods for calculating PRSs for SBP, DBP, and pulse pressure (PP): a weighted PRS limited to GWAS-significant single nucleotide polymorphisms (SNPs; i.e. those that fall below a *P*-value of 5 × 10^−8^), pruning and thresholding (P + T), and the two Bayesian regression frameworks LDpred,^21^ which assumes a point-normal mixture prior for the SNP effects, and PRS-CS,^20^ which uses continuous shrinkage (CS) priors. The SNP weights were derived from summary statistics of over 750 000 participants in a recent GWAS on BP that included over 1 million individuals of European ancestry.^11^ For the Bayesian approaches in LDpred and PRS-CS, a linkage disequilibrium (LD) reference panel from 1000 Genomes phase 3 version 5^19^ (*n* = 503) was used. The scores were developed and trained in the deCODE cohort, which contained a total of 1 024 311 overlapping genetic variants with the reference panel. HUNT and ALSPAC were used as testing cohorts, which were of similar ancestry to the training cohort.

For each PRS except for those limited to GWAS-significant SNPs, we tested a range of tuning parameters for each method to account for differences in the underlying genetic architecture between the traits, giving a total of 87 PRSs across all traits (see Supplementary material online, *Tables S1*–*S4*). As opposed to P + T, which utilizes LD-pruning and *P*-value thresholding, LDpred^21^ and PRS-CS^20^ assume a prior on SNP effects and account for LD using an external reference panel. The PRS with the highest correlation coefficient for each BP trait in the training cohort was identified and applied in downstream analysis.

### Phenotyping and outcome measures

Measurements of BP were obtained directly by trained personnel in all cohorts. In HUNT1, BP was assessed twice with a calibrated mercury sphygmomanometer after a period of seated rest. SBP was recorded at the first Korotkoff sound, and DBP was recorded at the disappearance of the fifth and final Korotkoff sound. In the subsequent surveys, participants were measured three times with an automated oscillometric device [Critikon Dinamap 845XT and 8100 (GE Medical Systems Information Technologies, Inc.) by the stationary team and XL9301 (Johnson & Johnson Medical, Inc.) by the mobile team in HUNT2-3; Dinamap Carescape V100 (GE Medical Systems Information Technologies, Inc.) by all teams in HUNT4].^30^ Individual BP was calculated as the mean of both HUNT1 measurements and the mean of the last two measurements in HUNT2-4. At deCODE, BP measurements and information about hypertension diagnoses were obtained from Landspitali—The National University Hospital of Iceland in Reykjavik, and the Primary Health Care Clinics of the Capital area, as well as at recruitment for deCODE studies, yielding BP measurements for 145 615 individuals with an average of 12 measurements per individual. A subset of 81 117 individuals with genotype information was used to calculate the PRSs. In ALSPAC, BP was measured twice at each time point. At ages 7, 9, and 11 years, the Dinamap 9301 Vital Signs Monitor (Morton Medical, London) was used; at age 10 years, the Omron MI-5 (Omron Healthcare, Kyoto, Japan) was used; at age 13 years, the Dinamap 8100 Vital Signs Monitor (Morton Medical) was used; and at ages 15 and 17 years, the Omron IntelliSense M6 (Omron Healthcare) was used.^31^ To adjust for the effects of antihypertensive medication in the adult cohorts, participants reporting use at the time of BP measurement, constants of 15 and 10 mmHg, were added to SBP and DBP, respectively, to control for pharmacological BP-lowering effects.^32^ Pulse pressure was calculated as the difference of the adjusted values (SBP—DBP).

Health registry data for HUNT participants were available from January of 1999 through March 2020. Disease outcomes were defined using the 10th revision of the International Statistical Classification of Diseases and Related Health Problems (ICD-10) and grouped into higher-level categories (see Supplementary material online, *Table S5*). Specifically, we combined relevant ICD-10 codes for cardiovascular disease (CVD), myocardial infarction (MI), chronic kidney disease (CKD), and stroke. Participants were considered hypertensive when I10 was registered in their medical records. For the single-cohort replication analysis, BP measurements were also used to establish hypertension status.

### Statistical analysis

Statistical analyses were performed using R (version 4.1.3) or Stata (version 16). We used linear spline multilevel models to test the predictive ability of the PRSs on the associated BP trait from birth and into early adulthood in ALSPAC using the top and bottom decile of the PRSs to represent high and low risks, respectively. The linear relationship between each PRS and BP trait was assessed cross-sectionally in each HUNT cohort and all cohorts (HUNT1-4) combined. This relationship was also investigated longitudinally within a subset of repeat participants (*n* = 18 498) with observations from all four surveys, from 1984 to 2019, stratified by low (1st PRS decile), average (2nd–9th PRS decile; reference), and high (10th PRS decile) genetic risk. Survival models were then created to determine time-to-event for BP-related outcomes as a function of genetic risk. We used Cox proportional hazards models (R package survival)^33^ to study the association between PRS and related disease outcomes among those with low, average, and high genetic risks. The models were adjusted for sex and the first 10 principal components, with age on the time scale. The proportional assumption of these models was verified graphically using log-minus-log plots,^34^ and the linearity assumption of the covariates was verified graphically using Martingale residual plots.^35^ Additionally, survival models with clinical risk factors alone and in combination with genetic data were compared using concordance indices and the likelihood ratio test. Polygenic risk scores performance for SBP was further assessed by replicating risk thresholds and stratification approaches used in similar studies. *P*-Values were interpreted as continuous indicators of evidence strength and conclusions were drawn based on effect sizes and their precision. Given the high correlation between BP traits, there was no correction for multiple testing.

## Results

**Figure 1:**
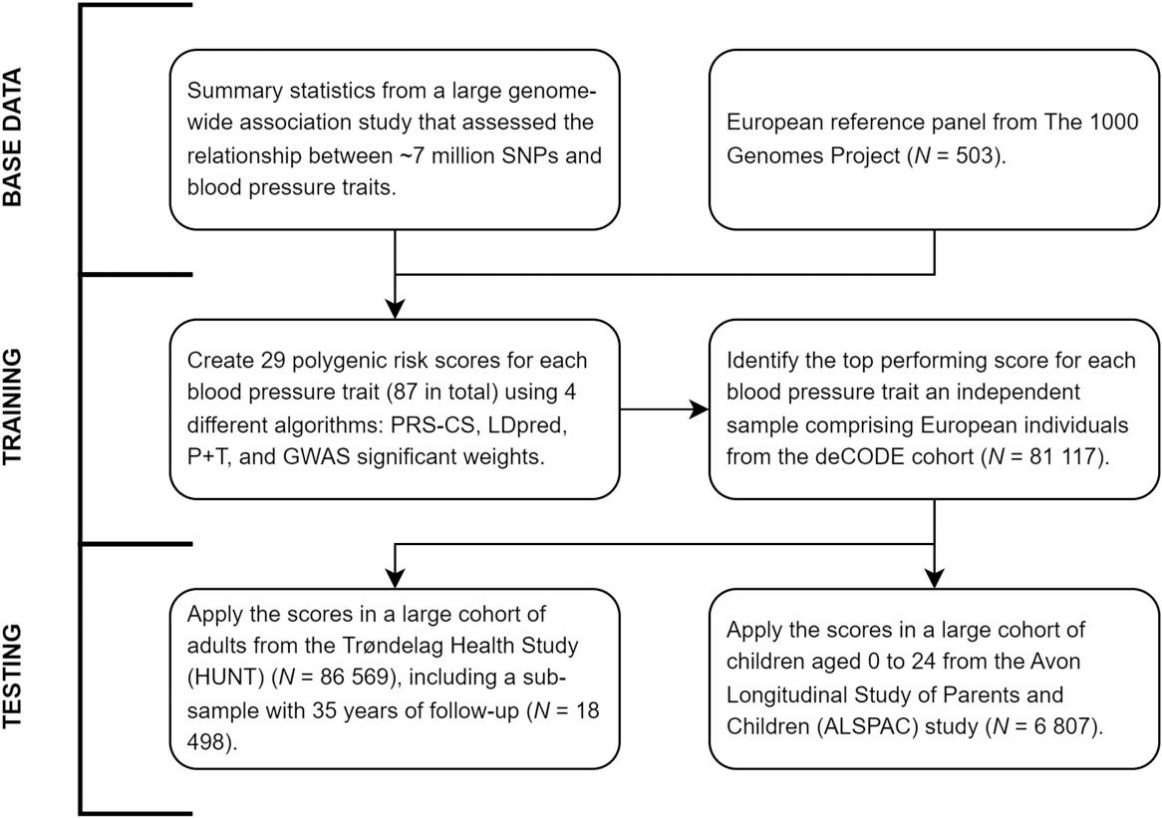
Polygenic risk score development for blood pressure traits. Effect sizes for single nucleotide polymorphism associated with blood pressure traits were obtained from the largest genome-wide association study to date by Evangelou *et al*.^11^ A cohort comprising genetic data from 503 Europeans participating in the 1000 Genomes Study^19^ was used as an external linkage disequilibrium reference panel. In the deCODE cohort, a total of 87 polygenic risk scores were created for systolic blood pressure, diastolic blood pressure, and pulse pressure using pruning and thresholding (P + T; 14 polygenic risk scores per trait), PRS-CS^20^ (6 polygenic risk scores per trait), and LDpred^21^ (8 polygenic risk scores per trait), with various tuning parameters, as well as a single weighted score using genome-wide association studies-significant single nucleotide polymorphisms. The best performing polygenic risk scores for each trait was then carried over to separate adult and children testing cohorts, from the Trøndelag Health Study (HUNT) and the Avon Longitudinal Study of Parents and Children, respectively.

After applying four different PRS methods to generate 87 scores with various tuning parameters across three BP traits in the deCODE cohort, we found that PRS-CS^20^ performed the best for all traits (see Supplementary material online, *Table S4*). Compared with the PRSs limited to GWAS-significant SNPs, the correlation coefficient for each BP trait improved by 47, 48, and 80% for SBP, DBP, and PP, respectively. These PRSs were subsequently carried over to independent testing cohorts (*Figure 1*).

### Associations between polygenic risk scores and blood pressure levels in childhood

**Figure 2:**
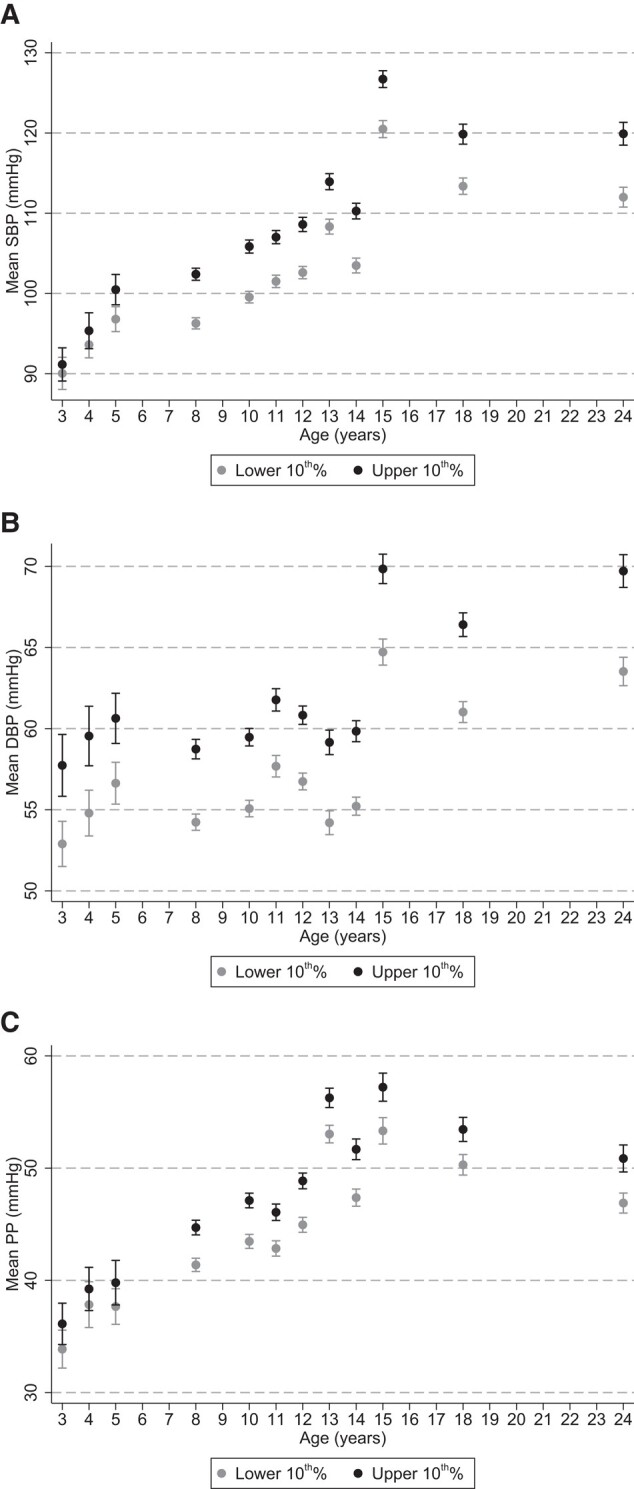
Polygenic risk scores and blood pressure trajectories in ALSPAC. Contains data from up to 6807 unique Avon Longitudinal Study of Parents and Children study participants between the ages of 3 and 24 years. Grey points represent the mean (and 95% confidence interval) blood pressure of participants in the lower 10th percentile of the polygenic risk score distribution and black points represent the mean (and 95% confidence interval) blood pressure of participants in the upper 10th percentile of the polygenic risk scores distribution.

Differences in BP between children with low and high PRS emerged early in life for all traits (*Figure 2*). A divergence in BP levels was observed at 3–5 years of age, with this difference becoming increasingly clear towards adulthood (see Supplementary material online, *Figures S1*–*S3*), resulting in a greater area below the BP curve (see Supplementary material online, *Figures S4*–*S6* and *Tables S8*–*S13*). Genetically susceptible children also appeared to exceed certain BP thresholds far earlier than those with the lowest genetic risk. For instance, an SBP of > 100 mmHg was observed in children with high PRS at 5 years of age, whereas their low-risk counterparts generally remained at < 100 mmHg until 11 years of age (*Figure 2*). Similarly, few children with low PRS ever exceeded a 65 mmHg DBP at any age, whereas all high-risk children were > 65 mmHg DBP, and some over 70 mmHg, from the age of 15.

### Associations between polygenic risk scores and blood pressure levels in adulthood

**Figure 3:**
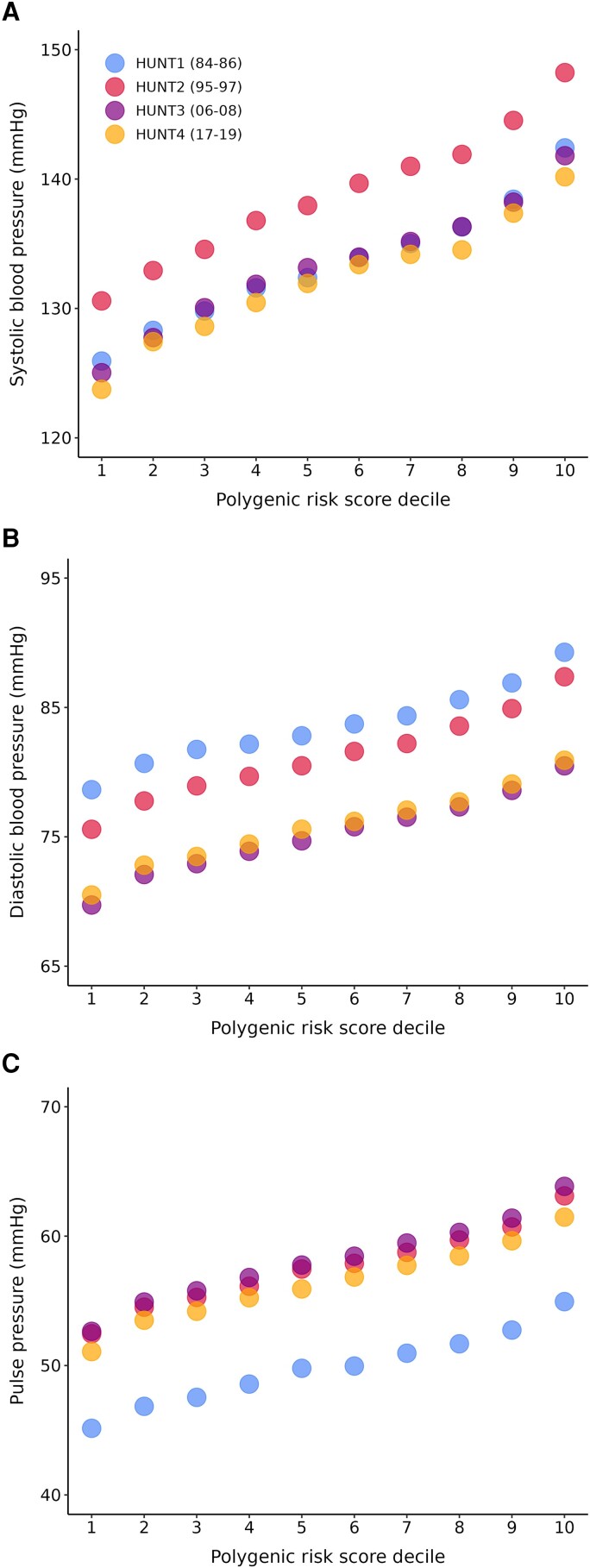
Blood pressure levels at each polygenic risk scores decile in HUNT. A total of 86 569 unique Trøndelag Health Study (HUNT) participants (cohort sample sizes with overlapping participation: 45 036 (HUNT1), 57 933 (HUNT2), 49 228 (HUNT3), and 53 148 (HUNT4); the years of data collection is in the brackets for each survey) were divided into polygenic risk score deciles for each blood pressure trait. The polygenic risk scores were derived using PRS-CS^20^ and adjusted for age and sex.

In the HUNT surveys, we observed linear relationships between all PRSs and their respective traits (*Figure 3*). The noticeably lower PP in all risk deciles in the earliest cohort (HUNT1, 1984–86) likely resulted from slightly lower and higher SBP and DBP, respectively, due to the participants being younger on average compared with previous surveys. The average increase in BP for each standard deviation change in the PRS was ∼5, 3, and 3 mmHg for SBP, DBP, and PP, respectively (see Supplementary material online, *Table S6*). Based on group means, those with a low PRS (1st decile) had a normal SBP (124–131 mmHg) and optimal DBP (71–80 mmHg); those with an average PRS (2nd–9th decile) had high normal SBP (132–139 mmHg) and normal to optimal DBP (75–83 mmHg); while those with a high PRS (10th decile) had an SBP in the hypertensive range (140–148 mmHg), with DBP spanning optimal to high normal (79–88 mmHg) (see Supplementary material online, *Table S7*).^4^ We performed survival analysis for early-onset (<55 years old) and late-onset (≥55 years old) hypertension, CVD, MI, CKD, and stroke. A high SBP PRS (top decile) substantially increased the risk of all adverse outcomes in HUNT (*Table 1*). Conversely, a low SBP PRS (bottom decile) reduced the risk of these outcomes.

**Table 1:** Morbidity and all-cause mortality risk at different levels of the polygenic risk scores in HUNT

	SBP PRS		DBP PRS		PP PRS	
	Low risk	High risk	Low risk	High risk	Low risk	High risk
Hypertension	0.52 (0.49, 0.56)	1.78 (1.71, 1.86)	0.58 (0.54, 0.61)	1.71 (1.64, 1.79)	0.68 (0.64, 0.72)	1.48 (1.42, 1.55)
Early-onset hypertension	0.35 (0.27, 0.46)	2.39 (2.14, 2.65)	0.45 (0.36, 0.57)	2.35 (2.11, 2.62)	0.71 (0.58, 0.85)	1.86 (1.65, 2.09)
Late-onset hypertension	0.55 (0.51, 0.58)	1.66 (1.58, 1.74)	0.59 (0.56, 0.63)	1.59 (1.52, 1.67)	0.69 (0.65, 0.73)	1.41 (1.35, 1.49)
Cardiovascular disease	0.80 (0.76, 0.83)	1.22 (1.18, 1.27)	0.83 (0.80, 0.87)	1.22 (1.17, 1.27)	0.85 (0.81, 0.88)	1.18 (1.14, 1.23)
Myocardial infarction	0.78 (0.71, 0.86)	1.33 (1.22, 1.46)	0.81 (0.73, 0.89)	1.22 (1.11, 1.34)	0.80 (0.72, 0.89)	1.21 (1.11, 1.33)
Stroke	0.78 (0.71, 0.85)	1.30 (1.19, 1.41)	0.79 (0.72, 0.87)	1.38 (1.27, 1.50)	0.81 (0.73, 0.89)	1.13 (1.04, 1.24)
Chronic kidney disease	0.74 (0.65, 0.85)	1.31 (1.16, 1.47)	0.81 (0.72, 0.92)	1.22 (1.08, 1.38)	0.74 (0.65, 0.85)	1.43 (1.28, 1.60)
All-cause mortality	0.83 (0.79, 0.88)	1.18 (1.12, 1.24)	0.84 (0.80, 0.88)	1.14 (1.08, 1.20)	0.87 (0.82, 0.91)	1.09 (1.04, 1.15)

PRS, polygenic risk score; HUNT, the Trøndelag Health Study; SBP, systolic blood pressure; DBP, diastolic blood pressure; PP, pulse pressure.

Early-onset hypertension: < 55 years old; late-onset hypertension: ≥ 55 years old. Hazard ratios (with 95% CIs) for low genetic risk (1st PRS decile) and high genetic risk (10th PRS decile) compared with average genetic risk (2nd–9th PRS decile). Disease follow-up between January 1999 and March 2020. All models were adjusted for sex and the first 10 principal components with age on the time scale.

To quantify the contribution of genetic data when added to traditional risk factors, we fitted and compared survival models for all disease outcomes consisting of either PRS only, clinical risk factors only, and both together. Variables in the traditional risk model were selected based on their established predictive ability for CVD and use in previous comparative analyses with BP PRSs,^17^ and included total and HDL cholesterol, SBP, use of antihypertensive medication, smoking, non-fasting blood glucose, and body mass index. All models were adjusted for sex, with age being used as time to event. Adding the PRSs improved model fit and concordance for all outcomes except early-onset hypertension (see Supplementary material online, *Tables S14* and *S15*). The unchanged concordance with this outcome may be due to the comparably small sample size below 55 years of age (*n* = 13 690) and consequent reduction in statistical power. When adding both SBP and DBP PRSs as covariates, the models further improved (see Supplementary material online, *Table S16*), with hypertension (2.57%) and CVD (1.69%) showing the greatest increases in concordance.

### Blood pressure trajectories in adulthood stratified by polygenic risk

**Figure 4:**
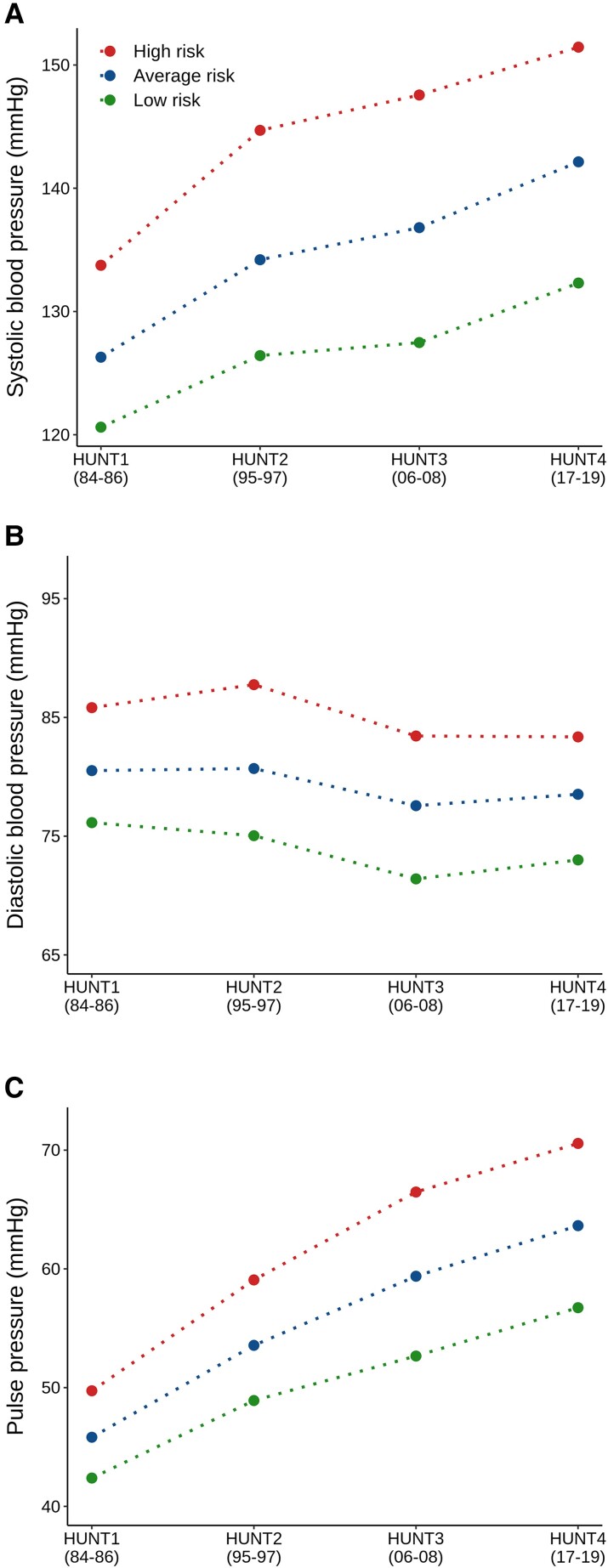
Polygenic risk scores and blood pressure trajectories in repeat HUNT participants. Blood pressure were obtained approximately every decade from 1984 to 2019 in a subset of 18 498 Trøndelag Health Study (HUNT) participants (56% female). Based on their polygenic risk score for each blood pressure trait, they were classified as low (1st polygenic risk scores decile), average (2nd–9th polygenic risk scores decile), or high (10th polygenic risk scores decile) risk.

To assess BP trajectories stratified by PRS in adulthood, we followed 18 498 repeat HUNT participants from 1985 to 2019 with follow-up each decade. Each risk stratum comprised 53, 56, and 56% women, for the low- (1st PRS decile), average- (2nd–9th PRS decile), and high-risk (10th decile) group, respectively. The mean age at the first survey for each level of genetic risk, as determined by SBP PRS, was 37.8 ± 9.4, 37.2 ± 9.3, and 36.9 ± 8.9 years, for low, average, and high risks, respectively. At this survey, these groups reported antihypertensive medication use of 0.3, 1.5, and 4.9%. At the end of follow-up, high-risk individuals reported approximately three times higher use of antihypertensives than low-risk individuals and yet had nearly 20 mmHg higher SBP (see Supplementary material online, *Table S17*). The estimated increases in SBP per HUNT survey across low- (1st decile), average- (2nd–9th decile), and high-risk (10th decile) individuals according to the PRS were 3.6 [95% confidence interval (CI): 1.0, 6.2] mmHg, 5.0 (95% CI: 2.0, 8.1) mmHg, and 5.1 (95% CI: −1.36, 11.6) mmHg, respectively (*Figure 4*). The BP slope did not differ between risk strata. The average increase for the whole subset was 4.6 (95% CI: −0.6, 9.7) mmHg.

### Disease risk according to genotype and phenotype

**Figure 5:**
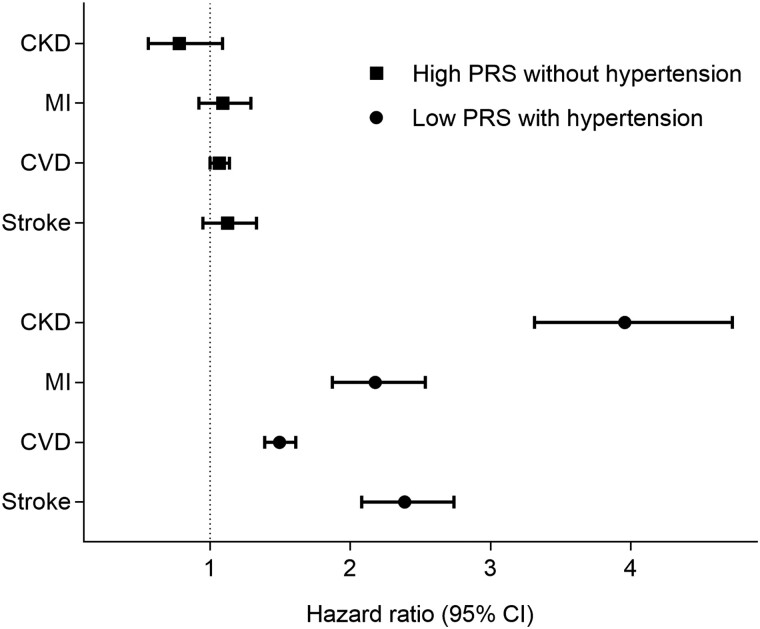
Morbidity risk with and without hypertension at different levels of genetic risk in HUNT. Risk of hypertension-related outcomes among individuals with a polygenic risk score for systolic blood pressure in the 10th decile without a hypertension diagnosis (*n* = 6243) compared with risk among participants in the Trøndelag Health Study (HUNT) in the first polygenic risk scores decile with a hypertension diagnosis (*n* = 1011). The second–ninth polygenic risk scores decile without a hypertension diagnosis (*n* = 55 999) used as reference. CKD, chronic kidney disease; MI, myocardial infarction; CVD, cardiovascular disease; CI, confidence intervals.

To explore whether a high genetic burden had implications for disease risk independent of phenotypic expression, we calculated hazard ratios (HRs) for individuals in the bottom and top decile of the SBP PRS distribution with and without a hypertension diagnosis, respectively, using average-risk individuals without hypertension as reference. Individuals in the lowest PRS decile with hypertension had a greater risk of all disease outcomes, while high PRS had equivalent risk to average PRS when a hypertension diagnosis was absent (*Figure 5*).

### Exploring different risk thresholds

A recent study in FinnGen and FINRISK participants found that individuals in the top 2.5% of the PRS distribution had a greater risk of and earlier onset of hypertension and cardiovascular outcomes.^17^ We performed replication analysis contrasting the top 2.5% of the risk distribution to those in the 20–80%. This analysis was limited to the HUNT4 cohort, which is comparable in time to the FinnGen population, and also allowed for the combination of measurement- and registry-based data to determine hypertension status without introducing additional bias. Similar risks in both cohorts were observed for all outcomes (see Supplementary material online, *Table S18*), with slightly higher HRs in our sample, indicating the robustness of this PRS approach. Based on sex differences in the genetic risk of hypertension, including both early- and late onset, in the same population,^36^ we also compared the risk of these outcomes for men and women separately using the same risk thresholds for the SBP PRS. Although the limited sample size for these comparisons together with the more stringent PRS threshold reduced the statistical power and thus ability to detect significant sex differences, men with a high PRS appeared to have lower HRs for all hypertension outcomes compared with their female counterparts (see Supplementary material online, *Table S19*). Conversely, men with a low PRS had higher HRs for the same outcomes compared with low PRS women.

## Discussion

We generated and tested 87 PRSs for SBP, DBP, and PP and found that a Bayesian regression framework with continuous shrinkage priors^20^ incorporating >1 million variants produced the strongest BP–PRS associations. Subsequent testing of these PRSs in children and adult cohorts showed that they were associated with both observed BP and hypertension-related disease risk and all-cause mortality. We found that individuals with high PRSs were at increased risk of cardiovascular and cerebrovascular diseases, as well as all-cause mortality, indicating that BP PRSs may have a clinically meaningful impact on risk identification and disease prevention. Moreover, we found that those with a low PRS and a hypertension diagnosis had higher disease risk than those with a high PRS in the absence of a hypertension diagnosis, indicating that phenotypic expression and not genetic susceptibility drives disease risk. This emphasizes the importance of BP management across the genetic risk spectrum.

The testing cohorts used in this study comprised both children and adults who underwent repeated BP measurements over several decades of study participation, which provided a unique opportunity to assess longitudinal PRS performance. Approximately 10% of children have elevated BP, and the prevalence is increasing.^37^ We observed that BP starts differing early in life between children with high and low PRS, with this relationship becoming increasingly clear towards adulthood, expanding upon previous findings.^31^ Similar differences were observed in adults, where all risk groups had a mean SBP within a range of ∼13 mmHg in their mid-30s, and differences in the rate of BP increase led to this range expanding to ∼19 mmHg in their early 70s. The early and continued exposure to elevated BP for genetically susceptible individuals reinforce the importance of early intervention to reduce the future burden of disease.^38,39^ This is supported by evidence showing that vascular injuries acquired at a young age appear to have greater consequences and lower plasticity than injuries of similar magnitude occurring later in life.^40–42^ A PRS can identify high-risk individuals who are likely to benefit from primordial prevention and its compounding effects throughout life.^43^

There is considerable overlap between SBP and DBP variants,^11,44^ with SNPs for both traits negatively affecting lifespan.^45^ Systolic BP is commonly observed to be a slightly better risk predictor than DBP,^7,46,47^ yet the PRS for both traits appeared to have a similar impact on risk in our adult testing cohort. Indeed, we found markedly different disease risks between low, average, and high PRS for all traits. While those with a low PRS had a reduced risk of developing hypertension both early and late in life, high-risk individuals had a considerably elevated risk of both, particularly early-onset hypertension. Additionally, the risk of hypertension-related diseases such as CVD, MI, stroke, and CKD was clearly higher in these individuals, likely related to a greater area under the BP curve due to a steeper increase in BP from birth to event.

In line with the probabilistic rather than deterministic nature of PRSs, we found that phenotypic expression appears more predictive of adverse health outcomes than the PRS alone. This is consistent with the previously reported risk-reducing properties of a healthy lifestyle at all levels of genetic susceptibility to coronary disease.^48^ Interestingly, for some cardiovascular phenotypes like familial hypercholesterolaemia, the genetic component may add to the inherent risk of pathological phenotypic expression, in this case, elevated LDL-cholesterol.^49,50^ For hypertension-related disease, however, we observed similar risk between those with high- and average PRS without a hypertension diagnosis, which was considerably lower than those with low PRS and hypertension. Although CVD risk is not fully captured by the conventional LDL-C measurement, this is also the case for a single BP measurement, which does not reflect BP throughout the day and can be prone to inaccuracies that affect its prognostic value.^51^ Clearly, both LDL-C and SBP are important treatment targets; their genetic underpinnings may, however, affect disease risk differently.

Some of the present findings are comparable to those by Vaura *et al*.,^17^ who recently reported similar results using the PRS-CS method^20^ to create BP PRSs in the Finnish population. Their comparison of the top 2.5% of SBP PRS carriers to the 20–80% range of the PRS distribution resulted in HRs (95% CI) of 1.30 (1.22, 1.39) and 1.29 (1.16, 1.44) for CVD and stroke, respectively. When matching these cut-offs in our sample, we observed HRs of 1.34 (1.19, 1.51) and 1.36 (1.01, 1.84), respectively. Additionally, we applied a broader definition for both the high-risk and average-risk categories and found HRs of 1.22 (1.18, 1.27) for CVD and 1.30 (1.19, 1.41) for stroke when comparing the top decile to the second through the ninth decile. A notable difference between our studies is that we were able to use the full summary statistics from ∼760 000 individuals in the largest BP GWAS^11^ for PRS development, whereas the overlap between this and the FINRISK cohorts restricted their base data sample size to ∼340 000 individuals.^17^ Although the HRs were higher for all outcomes in our sample, the differences were generally small, which could indicate that increasing the GWAS sample size beyond a certain level adds little predictive power to a PRS for BP traits.

To explore the assumption of sex-specific hypertension risk, we also replicated the sex-specific analyses by Kauko *et al*.^36^ In line with their findings, there appeared to be differences between sexes at both high and low PRS, although restricting our analyses to the HUNT4 survey resulted in limited statistical power. The estimated risk of hypertension during the lifespan, as well as both early- and late-onset hypertension, was lower in women in the <2.5% of the PRS distribution and higher in women in the >2.5% of the PRS distribution than their male counterparts, albeit with overlapping estimates between the groups. Ji *et al*.^52^ reported a steeper increase in BP in women compared with men, beginning in the third decade. This indicates sex-specific differences in BP-related phenotypes that interact with genetic factors to create distinct BP trajectories that ultimately may result in greater exposure to high BP among females and thus higher disease risk. This is further corroborated by findings showing that CVD risk occurs at a lower SBP for women than men.^53^ However, a caveat to sex-specific BP and its potential impact on disease risk is the difference in accuracy of BP measurement between sexes. Specifically, conventional measurements using a brachial cuff may underestimate true aortic BP in females.^54^ Thus, the sex differences in disease risk at a given BP may be partly due to inaccurate phenotyping.

Clinical screening for hypertension is recognized as an important step in preventing severe yet common health outcomes such as heart disease and stroke.^55^ Calculating PRSs, particularly for SBP, may be a low-cost, non-invasive approach to improve screening and thus BP control. Emerging evidence for cardiovascular risk factors such as SBP shows that the time course is a major factor in disease pathogenesis.^56^ Cumulative exposure to BP (mmHg × years) beginning in early adulthood is associated with increased risk of multiple cardiovascular and cerebrovascular outcomes.^57^ Additionally, inflammation induced by sustained elevated BP may cause irreversible changes in vascular and renal function.^58^ Thus, metrics that account for exposure time, such as time in the target range, may be preferable to discrete BP measurements to determine disease risk and degree of BP control.^59^

Evidence of the clinical utility of PRSs is encouraging yet equivocal.^60,61^ Some studies suggest that adding a PRS to traditional cardiovascular risk models adds little value,^62,63^ while others report improvements in traditional models after including genetic information.^17^ For most of the outcomes in the present study, combining traditional and genetic risk factors improved survival models compared with using either alone. The small magnitude of improvement must be interpreted in the context of traditional models relying on overexpressed risk factors, such as elevated BP or lipid values, to make estimations. While genetic data have limited predictive value in the presence of elevated clinical risk, which for many cardiovascular outcomes becomes increasingly common with age, they have the potential to indicate future disease risk from the beginning of life. The inverse association of disease risk and genetic susceptibility for all BP traits observed in the present study is a metric that is obtainable from birth. Conversely, a traditional model requires clinical risk factors, including age, to exceed a certain threshold value, limiting its effect on disease prevention. Moreover, these models can lead to systematic risk overestimation^64^ as well as misclassification of high-risk individuals.^65^ These are issues that gene-informed risk models are currently attempting to solve,^66^ but there are several challenges surrounding the return of genetic results.^67^ In addition to improving risk prediction, a PRS may also be able to identify less manageable hypertension phenotypes.^68^ This is in line with our findings, which showed that those with a high SBP PRS reported more frequent use of antihypertensive medication throughout life compared with low- and average-risk groups while still having substantially higher BP. Given the substantial inaccuracy of clinical BP measurements,^51^ genetic risk constructs can serve as additional indicators for patient follow-up.

With the increasing accuracy and non-invasiveness of polygenic predictors and decreasing cost of genotyping, arguments for the clinical use of PRSs for CVD and intermediate phenotypes such as BP and hypertension are growing stronger. Since the consequences of high BP generally occur after the reproductive years, BP-related SNPs are less susceptible to negative selection pressure and thus likely to remain prevalent in humans. Consequently, a considerable part of the population will remain at increased risk of developing hypertension, many of which could potentially benefit from insight into their genetic risk. Yet, the potential nocebo effects^69^ and unclear behavioural impact of communicating genetic risk to individuals^70^ must be acknowledged as potential barriers to clinical implementation.

The strengths of the present study include the use of large base, training, and testing datasets with no overlap; a direct performance comparison of novel and traditional PRS methods; the inclusion of longitudinal cohorts of children and adults, which provides new insights into the onset and lifelong progression of pathologically high BP in the context of a genetic predisposition; repeated BP measurement in a large subset from the mid-80s to the late 2010s; and registry-based disease follow-up. There were also several limitations, such as the adjustment for body mass index in the base data, which may increase the risk of collider bias;^11^ slight variation in the BP measurements related to instrument differences between surveys; selection bias in adult repeat HUNT participants; and reliance on self-reported medication use. Additionally, due to the number of participants who were surveyed more than once with decades between follow-up, combining BP measurements with registry data to create a measurement- and registry-based hypertension variable was challenging without introducing age bias. Thus, to avoid this, we limited our hypertension variable to a strictly registry-based diagnosis, which likely underestimates the number of true hypertension cases and, therefore the strength of the genotype-phenotype relationship. We did, however, perform replication analyses of a similar study^17^ using single-survey HUNT data where both measurements and registry data could be included in the outcome variable without introducing additional bias. Despite emerging findings with BP PRSs in other ancestries,^15,71^ there is still a discrepancy in the application of these and other PRSs in non-European cohorts.

The growing sample sizes of BP GWAS has increased the predictive power of various PRS methods. We characterized the impact of polygenic risk on BP traits and various disease outcomes from birth to old age using a novel risk quantification method and multiple independent cohorts. These genetic risk constructs may have important utility in a life-course treatment approach of high BP by leading to earlier identification of high-risk individuals and thereby earlier treatment, with subsequent reductions in the area under the BP curve and lifelong disease risk.

## Supplementary Material

zwad365_Supplementary_Data

## Data Availability

Researchers affiliated with a Norwegian research institution can apply for HUNT data access from HUNT Research Centre (www.ntnu.edu/hunt) if they have obtained project approval from the Regional Committee for Medical and Health Research Ethics (REC). Researchers not affiliated with a Norwegian research institution should collaborate with and apply through a Norwegian principal investigator. Information on the application and conditions for data access is available at www.ntnu.edu/hunt/data. The HUNT Databank website provides a detailed overview of the available variables in HUNT (www.ntnu.edu/hunt/databank). Certain data from ancillary HUNT projects may be subjected to a time-limited exclusivity provided to the researchers who have financed and conducted the data collection. Biologic material is available for analyses, and information on procedures is found at the HUNT-Biobank website (https://www.ntnu.edu/hunt/hunt-biobank). Data from the health registries are not kept by HUNT; instead, linkages between HUNT and registry data have to be made for each research project and require that the principal investigator has obtained project-specific approval for such linkage by REC and each registry owner. Requests to access ALSPAC data can be made by all researchers, independent of research area, institution, location, and funding source. The application process can be found online at http://www.bristol.ac.uk/alspac/researchers/access/.
