# Supplementary material for: Polygenic risk scores associate with blood pressure traits across the lifespan
Source: Eur J Prev Cardiol. 2023 Nov 26;31(6):644–54. doi: 10.1093/eurjpc/zwad365 (PMC11025038; doi:10.1093/eurjpc/zwad365)
Supplement: zwad365_Supplementary_Data [file zwad365_supplementary_data.zip › BP PRS - Supplements - R3 - clean.docx]

**SUPPLEMENTAL MATERIAL**

**Polygenic risk scores associate with blood pressure traits across the lifespan**

Table of contents

[Samples and genotyping 1](#_Toc140416662)

[Polygenic risk score construction 2](#_Toc140416663)

[Polygenic risk score performances 3](#_Toc140416664)

[Disease outcomes 7](#_Toc140416665)

[Results from HUNT 8](#_Toc140416666)

[Results from ALSPAC 9](#_Toc140416667)

[Tables 9](#_Toc140416668)

[Figures 15](#_Toc140416669)

[Comparison of genetic, clinical, and combined survival models 21](#_Toc140416670)

[Results from repeat HUNT participants 24](#_Toc140416671)

[Comparisons between HUNT and FinnGen 25](#_Toc140416672)

[References 26](#_Toc140416673)

# Samples and genotyping

The Trøndelag Health Study (HUNT) cohorts were genotyped using one of four different Illumina HumanCoreExome arrays (HumanCoreExome12 v1.0, HumanCoreExome12 v1.1, UM HUNT Biobank v1.0 and UM HUNT Biobank v2.0).^1^ Samples from HUNT1-3 were imputed using Minimac3 (v2.0.1, <http://genome.sph.umich.edu/wiki/Minimac3>)^2^ with default settings (2.5 Mb reference-based chunking with 500kb windows) and the HUNT-WGS customized Haplotype Reference consortium release 1.1 (HRC v1.1) for autosomal variants and HRC v1.1 for chromosome X variants.^3^ Samples from HUNT4 were phased with Eagle v2.4.1 (<https://alkesgroup.broadinstitute.org/Eagle/>) and imputed with the Positional Burrows-Wheeler Transform (PBWT) (<https://github.com/richarddurbin/pbwt>). More information about HUNT is available on the project’s website: [https://www.ntnu.edu/hunt](https://www.ntnu.edu/hunt/databank)

The deCODE cohort was characterized by whole-genome sequencing of 28 075 Icelanders using Illumina standard TruSeq methodology to a mean depth of 35× (SD 8×) with subsequent long-range phasing^4^ and imputing the information into 155 250 individuals chipped by multiple Illumina platforms.^5^ In total, up to 31.7 million variants have been characterized in this population. More information about deCODE is available on the project’s website: [https://www.decode.com/research](https://www.decode.com/research/)

The Avon Longitudinal Study of Parents and Children (ALSPAC) children cohort was genotyped using the Illumina HumanHap550 quad chip genotyping platforms by 23andMe subcontracting the Wellcome Trust Sanger Institute, Cambridge, UK and the Laboratory Corporation of America, Burlington, NC, US. Please note that the study website contains details of all the data that is available through a fully searchable data dictionary and variable search tool: <http://www.bristol.ac.uk/alspac/researchers/our-data/>

# Polygenic risk score construction

Summary statistics from the largest available blood pressure GWAS were used as a basis for deriving and comparing three popular polygenic risk score (PRS) methods: a weighted PRS limited to genome-wide significant single nucleotide polymorphisms (SNPs; i.e., those that fall below a p-value of 5×10^-8^), pruning and thresholding (P+T), and the two Bayesian regression frameworks LDpred,^6^ which assumes a point-normal mixture prior for the SNP effects, and PRS-CS,^7^ which uses continuous shrinkage priors. The Bayesian PRS methods were restricted to HapMap3 variants. Additionally, a score using only genome-wide significant variants (p < 5×10^-8^)^8^ was created. The scores were derived and trained in the deCODE cohort.^9^ The score with the highest correlation coefficient for each trait was identified and carried over for subsequent analyses in HUNT^1,10^ and ALSPAC,^11^ which acted as the testing sets.

The tools used to generate the different PRSs are available here: <https://github.com/getian107/PRScs> (PRS-CS); and here: <https://github.com/bvilhjal/ldpred> (LDpred). The latter package was also used in the creation of the P+T scores.

# Polygenic risk score performances

**Table S1.** Polygenic risk score performance for systolic blood pressure in deCODE

| **Method** | **Tuning parameter** | **N SNPs in score/N SNPs available** | **Pearson’s r** | **P-value** |
| --- | --- | --- | --- | --- |
| PRS-CS | 0.0001 | 1022624/1024311 (99.8%) | 0.192 | < 2e-16 |
| PRS-CS | 0.001 | 1022624/1024311 (99.8%) | 0.199 | < 2e-16 |
| PRS-CS | 0.01 | 1022624/1024311 (99.8%) | 0.199 | < 2e-16 |
| PRS-CS | 0.1 | 1022624/1024311 (99.8%) | 0.193 | < 2e-16 |
| PRS-CS | 1 | 1022624/1024311 (99.8%) | 0.186 | < 2e-16 |
| PRS-CS | Auto | 1022624/1024311 (99.8%) | 0.197 | < 2e-16 |
| LDpred | 1 | 1015377/1024311 (99.1%) | 0.197 | < 2e-16 |
| LDpred | 0.3 | 1015377/1024311 (99.1%) | 0.192 | < 2e-16 |
| LDpred | 0.1 | 1015377/1024311 (99.1%) | 0.168 | < 2e-16 |
| LDpred | 0.03 | 1015377/1024311 (99.1%) | 0.072 | < 2e-16 |
| LDpred | 0.01 | 1015377/1024311 (99.1%) | 0.031 | < 2e-16 |
| LDpred | 0.003 | 1015377/1024311 (99.1%) | 0.014 | 2.43e-11 |
| LDpred | 0.001 | 1015377/1024311 (99.1%) | 0.010 | 3.93e-4 |
| LDpred | Infinitesimal | 1015377/1024311 (99.1%) | 0.198 | < 2e-16 |
| P+T | r^2^ < 0.2 and p < 1 | 1015377/1024311 (99.1%) | 0.177 | < 2e-16 |
| P+T | r^2^ < 0.2 and p < 1e-1 | 275696/1024311 (26.9%) | 0.178 | < 2e-16 |
| P+T | r^2^ < 0.2 and p < 1e-2 | 112791/1024311 (11.0%) | 0.171 | < 2e-16 |
| P+T | r^2^ < 0.2 and p < 1e-3 | 59985/1024311 (5.9%) | 0.160 | < 2e-16 |
| P+T | r^2^ < 0.2 and p < 1e-4 | 37090/1024311 (3.6%) | 0.148 | < 2e-16 |
| P+T | r^2^ < 0.2 and p < 1e-5 | 25469/1024311 (2.5%) | 0.137 | < 2e-16 |
| P+T | r^2^ < 0.2 and p < 1e-6 | 18602/1024311 (1.8%) | 0.132 | < 2e-16 |
| P+T | r^2^ < 0.2 and p < 1e-7 | 14116/1024311 (1.4%) | 0.126 | < 2e-16 |
| P+T | r^2^ < 0.2 and p < 1e-8 | 11062/1024311 (1.1%) | 0.122 | < 2e-16 |
| P+T | r^2^ < 0.2 and p < 3e-1 | 482227/1024311 (47.1%) | 0.177 | < 2e-16 |
| P+T | r^2^ < 0.2 and p < 3e-2 | 165800/1024311 (16.2%) | 0.175 | < 2e-16 |
| P+T | r^2^ < 0.2 and p < 3e-3 | 79058/1024311 (7.7%) | 0.168 | < 2e-16 |
| P+T | r^2^ < 0.2 and p < 3e-4 | 46170/1024311 (4.5%) | 0.154 | < 2e-16 |
| P+T | r^2^ < 0.2 and p < 3e-5 | 30179/1024311 (2.9%) | 0.142 | < 2e-16 |
| GWAS | p < 5×10^-8^ | 860/868 (99.1%) | 0.135 | < 2e-16 |

**Table S2.** Polygenic risk score performance for diastolic blood pressure in deCODE

| **Method** | **Tuning parameter** | **N SNPs in score/N SNPs available** | **Pearson’s r** | **P-value** |
| --- | --- | --- | --- | --- |
| PRS-CS | 0.0001 | 1024311/1024311 (100%) | 0.206 | < 2e-16 |
| PRS-CS | 0.001 | 1024311/1024311 (100%) | 0.215 | < 2e-16 |
| PRS-CS | 0.01 | 1024311/1024311 (100%) | 0.215 | < 2e-16 |
| PRS-CS | 0.1 | 1024311/1024311 (100%) | 0.211 | < 2e-16 |
| PRS-CS | 1 | 1024311/1024311 (100%) | 0.204 | < 2e-16 |
| PRS-CS | Auto | 1024311/1024311 (100%) | 0.211 | < 2e-16 |
| LDpred | 1 | 1022462/1024311 (99.8%) | 0.195 | < 2e-16 |
| LDpred | 0.3 | 1022462/1024311 (99.8%) | 0.208 | < 2e-16 |
| LDpred | 0.1 | 1022462/1024311 (99.8%) | 0.184 | < 2e-16 |
| LDpred | 0.03 | 1022462/1024311 (99.8%) | 0.077 | < 2e-16 |
| LDpred | 0.01 | 1022462/1024311 (99.8%) | 0.031 | < 2e-16 |
| LDpred | 0.003 | 1022462/1024311 (99.8%) | 0.018 | 3.99e-09 |
| LDpred | 0.001 | 1022462/1024311 (99.8%) | 0.008 | < 1e-2 |
| LDpred | Infinitesimal | 1022462/1024311 (99.8%) | 0.209 | < 2e-16 |
| P+T | r^2^ < 0.2 and p < 1 | 1022462/1024311 (99.8%) | 0.193 | < 2e-16 |
| P+T | r^2^ < 0.2 and p < 1e-1 | 273923/1024311 (26.7%) | 0.195 | < 2e-16 |
| P+T | r^2^ < 0.2 and p < 1e-2 | 112597/1024311 (11.0%) | 0.189 | < 2e-16 |
| P+T | r^2^ < 0.2 and p < 1e-3 | 60450/1024311 (5.9%) | 0.179 | < 2e-16 |
| P+T | r^2^ < 0.2 and p < 1e-4 | 38260/1024311 (3.7%) | 0.171 | < 2e-16 |
| P+T | r^2^ < 0.2 and p < 1e-5 | 26504/1024311 (2.6%) | 0.162 | < 2e-16 |
| P+T | r^2^ < 0.2 and p < 1e-6 | 19241/1024311 (1.9%) | 0.155 | < 2e-16 |
| P+T | r^2^ < 0.2 and p < 1e-7 | 14823/1024311 (1.4%) | 0.148 | < 2e-16 |
| P+T | r^2^ < 0.2 and p < 1e-8 | 11631/1024311 (1.1%) | 0.143 | < 2e-16 |
| P+T | r^2^ < 0.2 and p < 3e-1 | 480671/1024311 (46.9%) | 0.193 | < 2e-16 |
| P+T | r^2^ < 0.2 and p < 3e-2 | 164685/1024311 (16.1%) | 0.193 | < 2e-16 |
| P+T | r^2^ < 0.2 and p < 3e-3 | 79094/1024311 (7.7%) | 0.184 | < 2e-16 |
| P+T | r^2^ < 0.2 and p < 3e-4 | 46586/1024311 (4.5%) | 0.174 | < 2e-16 |
| P+T | r^2^ < 0.2 and p < 3e-5 | 31389/1024311 (3.1%) | 0.166 | < 2e-16 |
| GWAS | p < 5×10^-8^ | 860/868 (99.1%) | 0.145 | < 2e-16 |

**Table S3.** Polygenic risk score performance for pulse pressure in deCODE

| **Method** | **Tuning parameter** | **N SNPs in score/N SNPs available** | **Pearson’s r** | **P-value** |
| --- | --- | --- | --- | --- |
| PRS-CS | 0.0001 | 1022643/1024311 (99.8%) | 0.173 | < 2e-16 |
| PRS-CS | 0.001 | 1022643/1024311 (99.8%) | 0.178 | < 2e-16 |
| PRS-CS | 0.01 | 1022643/1024311 (99.8%) | 0.173 | < 2e-16 |
| PRS-CS | 0.1 | 1022643/1024311 (99.8%) | 0.166 | < 2e-16 |
| PRS-CS | 1 | 1022643/1024311 (99.8%) | 0.158 | < 2e-16 |
| PRS-CS | Auto | 1022643/1024311 (99.8%) | 0.175 | < 2e-16 |
| LDpred | 1 | 1015376/1024311 (99.1%) | 0.168 | < 2e-16 |
| LDpred | 0.3 | 1015376/1024311 (99.1%) | 0.172 | < 2e-16 |
| LDpred | 0.1 | 1015376/1024311 (99.1%) | 0.145 | < 2e-16 |
| LDpred | 0.03 | 1015376/1024311 (99.1%) | 0.069 | < 2e-16 |
| LDpred | 0.01 | 1015376/1024311 (99.1%) | 0.031 | < 2e-16 |
| LDpred | 0.003 | 1015376/1024311 (99.1%) | 0.011 | 7.03e-07 |
| LDpred | 0.001 | 1015376/1024311 (99.1%) | 0.005 | 2.46e-2 |
| LDpred | Infinitesimal | 1015376/1024311 (99.1%) | 0.171 | < 2e-16 |
| P+T | r^2^ < 0.2 and p < 1 | 1015376/1024311 (99.1%) | 0.156 | < 2e-16 |
| P+T | r^2^ < 0.2 and p < 1e-1 | 256004/1024311 (25.0%) | 0.160 | < 2e-16 |
| P+T | r^2^ < 0.2 and p < 1e-2 | 98125 /1024311 (9.6%) | 0.156 | < 2e-16 |
| P+T | r^2^ < 0.2 and p < 1e-3 | 50234/1024311 (4.9%) | 0.147 | < 2e-16 |
| P+T | r^2^ < 0.2 and p < 1e-4 | 30809/1024311 (3.0%) | 0.138 | < 2e-16 |
| P+T | r^2^ < 0.2 and p < 1e-5 | 21022/1024311 (2.1%) | 0.133 | < 2e-16 |
| P+T | r^2^ < 0.2 and p < 1e-6 | 15606/1024311 (1.5%) | 0.126 | < 2e-16 |
| P+T | r^2^ < 0.2 and p < 1e-7 | 11774/1024311 (1.1%) | 0.119 | < 2e-16 |
| P+T | r^2^ < 0.2 and p < 1e-8 | 9078/1024311 (0.9%) | 0.116 | < 2e-16 |
| P+T | r^2^ < 0.2 and p < 3e-1 | 462985/1024311 (45.2%) | 0.158 | < 2e-16 |
| P+T | r^2^ < 0.2 and p < 3e-2 | 148809/1024311 (14.5%) | 0.160 | < 2e-16 |
| P+T | r^2^ < 0.2 and p < 3e-3 | 67100 /1024311 (6.6%) | 0.151 | < 2e-16 |
| P+T | r^2^ < 0.2 and p < 3e-4 | 38258/1024311 (3.7%) | 0.141 | < 2e-16 |
| P+T | r^2^ < 0.2 and p < 3e-5 | 24975 /1024311 (2.4%) | 0.134 | < 2e-16 |
| GWAS | p < 5×10^-8^ | 253/256 (98.8%) | 0.099 | < 2e-16 |

**Table S4.** The best performing PRSs from each method for each trait in deCODE

| **Method** | **Tuning parameter** | **Trait** | **N SNPs in score/N SNPs available** | **Pearson’s r** | **P-value** |
| --- | --- | --- | --- | --- | --- |
| PRS-CS | 0.001 | SBP | 1022624/1024311 (99.8%) | 0.199 | < 2e-16 |
| LDpred | Infinitesimal | SBP | 1015377/1024311 (99.1%) | 0.198 | < 2e-16 |
| P+T | r^2^ < 0.2 and p < 0.1 | SBP | 275696/1024311 (26.9%) | 0.178 | < 2e-16 |
| GWAS | p < 5×10^-8^ | SBP | 860/868 (99.1%) | 0.135 | < 2e-16 |
| PRS-CS | 0.01 | DBP | 1024311/1024311 (100%) | 0.215 | < 2e-16 |
| LDpred | Infinitesimal | DBP | 1022462/1024311 (99.8%) | 0.209 | < 2e-16 |
| P+T | r^2^ < 0.2 and p < 0.1 | DBP | 273923/1024311 (26.7%) | 0.195 | < 2e-16 |
| GWAS | p < 5×10^-8^ | DBP | 860/868 (99.1%) | 0.145 | < 2e-16 |
| PRS-CS | 0.001 | PP | 1022643/1024311 (99.8%) | 0.178 | < 2e-16 |
| LDpred | 0.3 | PP | 1015376/1024311 (99.1%) | 0.172 | < 2e-16 |
| P+T | r^2^ < 0.2 and p < 0.03 | PP | 148809/1024311 (14.5%) | 0.160 | < 2e-16 |
| GWAS | p < 5×10^-8^ | PP | 253/256 (98.8%) | 0.099 | < 2e-16 |

*PRS, polygenic risk score; SNP, single nucleotide polymorphism; SBP, systolic blood pressure; DBP, diastolic blood pressure; PP, pulse pressure.*

# Disease outcomes

Systolic and diastolic blood pressure (SBP and DBP, respectively) were measured directly in all participants and adjusted for the use of medication, where applicable. Pulse pressure (PP) was calculated based on SBP and DBP measurements. Hypertension, cardiovascular disease (CVD), myocardial infarction (MI), stroke, and chronic kidney disease (CKD) were defined using the 10th revision of the International Statistical Classification of Diseases and Related Health Problems (ICD-10) (**Table S5**).

**Table S5.** Definition of outcomes using the 10th revision of the International Statistical Classification of Diseases and Related Health Problems (ICD-10)

| Outcome | ICD-10 code(s) |
| --- | --- |
| Hypertension | I10 |
| Cardiovascular disease | I11.0, I11.9, I13.0, I13.1, I13.2, I13.9, I20.0, I20.1, I20.8, I20.9, I21.0, I21.01, I21.02, I21.03, I21.0a, I21.0b, I21.1, I21.11, I21.13, I21.1a, I21.2, I21.21, I21.3, I21.31, I21.32, I21.3b, I21.4, I21.41, I21.42, I21.4a, I21.4b, I21.9, I21.91, I21.92, I21.9a, I24.8, I24.9, I25.0, I25.1, I25.2, I25.3, I25.4, I25.5, I25.6, I25.8, I25.9,I26.0, I26.9, I27.0, I27.2, I27.8, I27.9, I34.0, I34.1, I34.2, I34.8, I34.9, I35.0, I35.1, I35.2, I35.8, I35.9, I36.0, I36.1, I36.2, I36.8, I36.9, I37.0, I37.1, I37.8, I37.9, I42.0, I42.1, I42.2, I42.9, I44.0, I44.1, I44.2, I44.3, I44.4, I44.5, I44.6, I44.7, I45.0, I45.1, I45.2, I45.3, I45.4, I45.5, I45.6, I45.8, I45.9, I46.0, I46.1, I46.9, I47.0, I47.1, I47.2, I47.9, I48, I48.0, I48.1, I48.2, I48.3, I48.4, I48.9, I49.0, I49.1, I49.2, I49.3, I49.4, I49.5, I49.8, I49.9, I50.0, I50.1, I50.9, I51.0, I51.1, I51.2, I51.3, I51.4, I51.5, I51.6, I51.7, I51.8, I51.9, I60.0, I60.1, I60.2, I60.3, I60.4, I60.5, I60.6, I60.7, I60.8, I60.9, I61.0, I61.1, I61.2, I61.3, I61.4, I61.5, I61.6, I61.8, I61.9, I62.0, I62.1, I62.9, I63.0, I63.1, I63.2, I63.3, I63.4, I63.5, I63.6, I63.8 I63.9, I64, I65.0, I65.1, I65.2, I65.3, I65.8, I65.9, I66.0, I66.1, I66.2, I66.3, I66.4, I66.8, I66.9, I67.0, I67.1, I67.2, I67.3, I67.4, I67.5, I67.6, I67.7, I67.8, I67.9, I68.0, I70.0, I70.00, I70.01, I70.1, I70.10, I70.2, I70.20, I70.21, I70.8, I70.80, I70.81, I70.9, I70.90, I70.91, I71.0, I71.1, I71.2, I71.3, I71.4, I71.5, I71.6, I71.8, I71.9, I72.0, I72.1, I72.2, I72.3, I72.4, I72.5, I72.6, I72.8, I72.9, I73.9, I74.0, I74.1, I74.2, I74.3, I74.4, I74.5, I74.8, I74.9, I82.2, I82.3, I82.8, I82.9 |
| Myocardial infarction | I21.0, I21.01, I21.02, I21.03, I21.0a, I21.0b, I21.1, I21.11, I21.13, I21.1a, I21.2, I21.21, I21.3, I21.31, I21.32, I21.3b, I21.4, I21.41, I21.42, I21.4a, I21.4b, I21.9, I21.91, I21.92, I21.9a |
| Stroke | I60.0, I60.1, I60.2, I60.3, I60.4, I60.5, I60.6, I60.7, I60.8, I60.9, I61.0, I61.1, I61.2, I61.3, I61.4, I61.5, I61.6, I61.8, I61.9, I62.0, I62.1, I62.9, I63.0, I63.1, I63.2, I63.3, I63.4, I63.5, I63.6, I63.8, I63.9, I64 |
| Chronic kidney disease | I12.0, I12.9, I13.0, I13.1, I13.2, I13.9, N18.0, N18.1, N18.2, N18.3, N18.4, N18.5, N18.8, N18.9 |

# Results from HUNT

**Table S6.** Correlation coefficients for PRSs and BP traits in HUNT

| **Survey and trait** | **Pearson’s r** | **P-value** | **↑ mmHg (95% CI) per PRS SD** |
| --- | --- | --- | --- |
| **HUNT1** |  |  |  |
| SBP | 0.227 | < 2.2e-16 | 4.7 (4.5 – 4.9) |
| DBP | 0.252 | < 2.2e-16 | 3.0 (2.8 – 3.1) |
| PP | 0.190 | < 2.2e-16 | 2.8 (2.7 – 2.9) |
| **HUNT2** |  |  |  |
| SBP | 0.222 | < 2.2e-16 | 5.1 (5.0 – 5.3) |
| DBP | 0.254 | < 2.2e-16 | 3.3 (3.2 – 3.4) |
| PP | 0.195 | < 2.2e-16 | 3.0 (2.9 – 3.1) |
| **HUNT3** |  |  |  |
| SBP | 0.229 | < 2.2e-16 | 4.7 (4.6 – 4.9) |
| DBP | 0.252 | < 2.2e-16 | 3.1 (3.0 – 3.2) |
| PP | 0.216 | < 2.2e-16 | 3.1 (3.0 – 3.3) |
| **HUNT4** |  |  |  |
| SBP | 0.223 | < 2.2e-16 | 4.7 (4.5 – 4.9) |
| DBP | 0.264 | < 2.2e-16 | 3.0 (2.9 – 3.1) |
| PP | 0.203 | < 2.2e-16 | 3.0 (2.9 – 3.1) |

*PRS, polygenic risk score; BP, blood pressure; HUNT, the Trøndelag Health Study; SBP, systolic blood pressure; DBP, diastolic blood pressure; PP, pulse pressure; CI, confidence intervals; SD, standard deviation.*

**Table S7.** Mean adjusted SBP/DBP at each level of PRS in HUNT

|  | **Low PRS** | **Average PRS** | **High PRS** | ***N*** | **Age** | **Female %** |
| --- | --- | --- | --- | --- | --- | --- |
| HUNT1 | 126/80 | 133/83 | 142/88 | 45 036 | 43.0 ± 14.1 | 53% |
| HUNT2 | 131/77 | 139/81 | 148/86 | 57 933 | 49.5 ± 16.8 | 53% |
| HUNT3 | 125/71 | 133/75 | 142/79 | 49 228 | 53.2 ± 16.0 | 55% |
| HUNT4 | 124/71 | 132/76 | 140/80 | 53 148 | 55.4 ± 17.7 | 55% |

*SBP, systolic blood pressure; DBP, diastolic blood pressure; PRS, polygenic risk score; HUNT, the Trøndelag Health Study.*

# Results from ALSPAC

## Tables

**Table S8.** Summary of measurements included in the model and model fit for SBP (mmHg) trajectories

|  | **Summary of Measurements** | | |  | **Model fit for SBP trajectories** | | |
| --- | --- | --- | --- | --- | --- | --- | --- |
|  | **Number of participants with at least one measure of SBP^1^** | **Total number of measures** | **Median (IQR) measures per participant** | **Mean (SD) predicted intercept and slopes^2^** | **Mean predicted SBP in mmHg (SD)^3^** | **Mean observed SBP in mmHg (SD)** | **Mean difference between observed and predicted SBP in mmHg (SD)^4^** |
| Overall | 6807 | 38801 | 7 (6 to 8) | - | - | - | - |
| 3 years | 704 | 704 | 1 (1 to 1) | 90.46 (5.16) | 90.46 (5.17) | 90.16 (8.10) | 0.12 (-11.76, 11.79) |
| 3 years – 5 years | 770 | 1748 | 3 (2 to 3) | 4.15 (1.53) | 93.78 (5.80) | 93.50 (8.43) | -0.16 (-11.65, 11.33) |
| 5 years – 7 years | 4536 | 4744 | 1 (1 to 1) | -0.19 (1.07) | 98.90 (5.92) | 98.68 (8.97) | 0.16 (-9.97, 10.28) |
| 7 years – 12 years | 6163 | 14990 | 3 (2 to 3) | 1.61 (0.40) | 103.58 (6.33) | 103.42 (9.43) | -0.08 (-12.40, 12.24) |
| 12 years – 15 years | 5374 | 9684 | 2 (2 to 2) | 5.18 (1.35) | 112.46 (8.76) | 112.52 (12.67) | 0.57 (-13.86, 15.00) |
| 15 years – 24 years | 4368 | 7635 | 2 (2 to 2) | -0.63 (0.36) | 118.21 (8.20) | 117.41 (11.38) | -0.61 (-14.30, 13.08) |

*Data presented as mean and standard deviation (SD).*

*^1^Individuals who had full data on systolic blood pressure (SBP) and were in the sequence set.*

*^2^The data at 3 years relate to predictions from the multilevel model at exactly 3 years (i.e., the intercept).
^3^The data at 3 years relate to the SBP measurement carried out at a mean age of 3.08 years.
^4^Range within which 95% of the differences between observed SBP measurements and those predicted by the multilevel model line.*

**Table S9.** Association between the polygenic risk score with predicted SBP trajectory between the ages of 3 years and 24 years using linear spline multilevel models

| **Intercept and slopes** | **Mean SBP trajectory (95% CI) in reference group^1^** | **Difference in the intercept and slopes between ages with the PRS (upper 10^th^ vs. lower 10^th^ percentile)^2^** | |
| --- | --- | --- | --- |
|  |  | **Estimate (95% CI)** | **P-value** |
| 3 years (mmHg) | 88.33 (86.40, 90.26) | 1.98 (-0.21, 4.17) | 0.08 |
| Change between 3 years – 5 years (mmHg/year) | 4.21 (2.93, 5.48) | 1.12 (-0.30, 2.55) | 0.12 |
| Change between 5 years – 7 years (mmHg/year) | -0.58 (-1.52, 0.36) | 1.03 (-0.03, 2.08) | 0.06 |
| Change between 7 years – 12 years (mmHg/year) | 1.69 (1.47, 1.92) | -0.13 (-0.40, 0.13) | 0.32 |
| Change between 12 years – 15 years (mmHg/year) | 4.02 (3.63, 4.41) | 0.12 (-0.35, 0.58) | 0.62 |
| Change between 15 years – 24 years (mmHg/year) | -0.98 (-1.13, -0.83) | 0.15 (-0.04, 0.34) | 0.12 |

*Polygenic risk score (PRS) defined as a binary variable with the bottom and top decile of the risk distribution; SBP, systolic blood pressure; CI, confidence intervals.*

*^1^The reference group included individuals in the lowest decile of the PRS.*

*^2^Adjusted for sex.*

**Table S10.** Summary of measurements included in the model and model fit for DBP (mmHg) trajectories

|  | **Summary of Measurements** | | |  | **Model fit for DBP trajectories** | | |
| --- | --- | --- | --- | --- | --- | --- | --- |
|  | **Number of participants with at least one measure of DBP^1^** | **Total number of measures** | **Median (IQR) measures per participant** | **Mean (SD) predicted intercept and slopes^2^** | **Mean predicted DBP in mmHg (SD)^3^** | **Mean observed DBP in mmHg (SD)** | **Mean difference between observed and predicted DBP in mmHg (SD)^4^** |
| Overall | 6807 | 38803 | 7 (6 to 8) | - | - | - | - |
| 3 years | 704 | 704 | 1 (1 to 1) | 54.93 (4.21) | 54.93 (4.21) | 55.74 (6.39) | -0.05 (-8.81, 8.71) |
| 3 years – 5 years | 770 | 1749 | 3 (2 to 3) | 1.69 (0.69) | 56.39 (4.21) | 56.94 (6.35) | -0.10 (-9.18, 8.99) |
| 5 years – 7 years | 4534 | 4742 | 1 (1 to 1) | -0.93 (1.04) | 56.85 (4.11) | 56.58 (6.65) | -0.15 (-7.90, 7.60) |
| 7 years – 12 years | 6163 | 14993 | 3 (2 to 3) | 0.25 (0.34) | 57.43 (3.68) | 58.44 (7.09) | 0.84 (-9.97, 11.66) |
| 12 years – 15 years | 5374 | 9684 | 2 (2 to 2) | 2.37 (0.38) | 60.69 (4.45) | 59.58 (9.31) | -0.93 (-14.35, 12.49) |
| 15 years – 24 years | 4368 | 7635 | 2 (2 to 2) | 0.12 (0.17) | 65.52 (3.97) | 65.58 (7.60) | -0.11 (-11.87, 11.66) |

*Data presented as mean and standard deviation (SD).*

*^1^Individuals who had full data on diastolic blood pressure (DBP) and were in the sequence set.*

*^2^The data at 3 years relate to predictions from the multilevel model at exactly 3 years (i.e., the intercept).
^3^The data at 3 years relate to the DBP measurement carried out at a mean age of 3.08 years.
^4^Range within which 95% of the differences between observed DBP measurements and those predicted by the multilevel model line.*

**Table S11.** Association between the polygenic risk score with predicted DBP trajectory between the ages of 3 years and 24 years using linear spline multilevel models

| **Intercept and slopes** | **Mean DBP trajectory (95% CI) in reference group^1^** | **Difference in the intercept and slopes between ages with the PRS (upper 10^th^ vs. lower 10^th^ percentile)^2^** | |
| --- | --- | --- | --- |
|  |  | **Estimate (95% CI)** | **P-value** |
| 3 years (mmHg) | 52.42 (50.78, 54.06) | 4.64 (2.73, 6.55) | 1.84x10^-06^ |
| Change between 3 years – 5 years (mmHg/year) | 2.21 (1.09, 3.33) | -0.82 (-2.12, 0.48) | 0.22 |
| Change between 5 years – 7 years (mmHg/year) | -0.95 (-1.80, -0.11) | 0.78 (-0.19, 1.76) | 0.12 |
| Change between 7 years – 12 years (mmHg/year) | 0.20 (0.02, 0.38) | -0.09 (-0.30, 0.12) | 0.41 |
| Change between 12 years – 15 years (mmHg/year) | 2.21 (1.89, 2.53) | 0.27 (-0.10, 0.65) | 0.16 |
| Change between 15 years – 24 years (mmHg/year) | 0.07 (-0.08, 0.22) | 0.10 (-0.08, 0.28) | 0.27 |

*Polygenic risk score (PRS) defined as a binary variable with the bottom and top decile of the risk distribution; DBP, diastolic blood pressure; CI, confidence intervals.*

*^1^The reference group included individuals in the lowest decile of the PRS*

*^2^Adjusted for sex*

**Table S12.** Summary of measurements included in the model and model fit for PP (mmHg) trajectories

|  | **Summary of Measurements** | | |  | **Model fit for PP trajectories** | | |
| --- | --- | --- | --- | --- | --- | --- | --- |
|  | **Number of participants with at least one measure of PP^1^** | **Total number of measures** | **Median (IQR) measures per participant** | **Mean (SD) predicted intercept and slopes^2^** | **Mean predicted PP in mmHg (SD)^3^** | **Mean observed PP in mmHg (SD)** | **Mean difference between observed and predicted PP in mmHg (SD)^4^** |
| Overall | 6807 | 38799 | 7 (6 to 8) | - | - | - | - |
| 3 years | 704 | 704 | 1 (1 to 1) | 35.36 (3.40) | 35.36 (3.40) | 34.41 (7.19) | -0.52 (-13.80, 12.77) |
| 3 years – 5 years | 770 | 1748 | 3 (2 to 3) | 1.96 (1.02) | 36.86 (3.64) | 36.56 (7.53) | 0.23 (-12.22, 12.68) |
| 5 years – 7 years | 4534 | 4742 | 1 (1 to 1) | 1.12 (0.85) | 41.97 (4.17) | 42.10 (7.81) | 0.31 (-10.72, 11.35) |
| 7 years – 12 years | 6163 | 14990 | 3 (2 to 3) | 1.38 (0.19) | 46.15 (4.39) | 44.99 (7.91) | -0.97 (-12.95, 11.02) |
| 12 years – 15 years | 5374 | 9684 | 2 (2 to 2) | 2.52 (1.51) | 51.43 (5.95) | 52.94 (10.52) | 1.58 (-13.17, 16.34) |
| 15 years – 24 years | 4368 | 7635 | 2 (2 to 2) | -0.71 (0.48) | 51.99 (7.05) | 51.83 (10.14) | -0.52 (-14.37, 13.33) |

*Data presented as mean and standard deviation (SD).*

*^1^Individuals who had full data on pulse pressure (PP) and were in the sequence set.*

*^2^The data at 3 years relate to predictions from the multilevel model at exactly 3 years (i.e., the intercept).
^3^The data at 3 years relate to the PP measurement carried out at a mean age of 3.08 years.*

**Table S13.** Association between the polygenic risk score with predicted PP trajectory between the ages of 3 years and 24 years using linear spline multilevel models

| **Intercept and slopes** | **Mean PP trajectory (95% CI) in reference group^1^** | **Difference in the intercept and slopes between ages with the PRS (upper 10^th^ vs. lower 10^th^ percentile)^2^** | |
| --- | --- | --- | --- |
|  |  | **Estimate (95% CI)** | **P-value** |
| 3 years (mmHg) | 33.55 (31.98, 35.12) | 2.01 (0.12, 3.91) | 0.04 |
| Change between 3 years – 5 years (mmHg/year) | 2.63 (1.33, 3.93) | -0.06 (-1.58, 1.45) | 0.93 |
| Change between 5 years – 7 years (mmHg/year) | 0.31 (-0.70, 1.32) | 0.75 (-0.42, 1.91) | 0.21 |
| Change between 7 years – 12 years (mmHg/year) | 1.45 (1.26, 1.65) | 0.07 (-0.16, 0.29) | 0.56 |
| Change between 12 years – 15 years (mmHg/year) | 1.55 (1.16, 1.94) | -0.02 (-0.49, 0.45) | 0.94 |
| Change between 15 years – 24 years (mmHg/year) | -0.91 (-1.06, -0.77) | -0.01 (-0.19, 0.17) | 0.92 |

*Polygenic risk score (PRS) defined as a binary variable with the bottom and top decile of the risk distribution; PP, pulse pressure; CI, confidence intervals.*

*^1^The reference group included individuals in the lowest decile of the PRS.*

*^2^Adjusted for sex*

## Figures

**Figure S1.** Association between the polygenic risk score and SBP at age 3, 14 and 24 years

*Points (and lines) represent the mean systolic blood pressure (SBP) (and 95% confidence interval) of participants across deciles of the SBP polygenic risk score distribution at age 3 (earliest measure), 14 (mid-point measure) and 24 (latest measure) years.*

**Figure S2.** Association between the polygenic risk score and DBP at age 3, 14 and 24 years

*Points (and lines) represent the mean diastolic blood pressure (DBP) (and 95% confidence interval) of participants across deciles of the DBP polygenic risk score distribution at age 3 (earliest measure), 14 (mid-point measure) and 24 (latest measure) years.*

**Figure S3.** Association between the polygenic risk score and PP at age 3, 14 and 24 years

*Points (and lines) represent the mean pulse pressure (PP) (and 95% confidence interval) of participants across deciles of the PP polygenic risk score distribution at age 3 (earliest measure), 14 (mid-point measure) and 24 (latest measure) years.*

**Figure S4.** Association between the polygenic risk score and SBP trajectory between the ages of 3 years and 24 years using linear spline multilevel models

*Values for the lowest decile and highest decile of the systolic blood pressure (SBP) polygenic risk score are depicted in light and darker grey, respectively.*

**Figure S5.** Association between the polygenic risk and DBP trajectory between the ages of 3 years and 24 years using linear spline multilevel models

*Values for the lowest decile and highest decile of the diastolic blood pressure (DBP) polygenic risk score are depicted in light and darker grey, respectively.*

**Figure S6.** Association between the polygenic risk score and PP trajectory between the ages of 3 years and 24 years using linear spline multilevel models

*Values for the lowest decile and highest decile of the pulse pressure (PP) polygenic risk score are depicted in light and darker grey, respectively.*

# Comparison of genetic, clinical, and combined survival models

**Table S14.** Model comparison with SBP PRS

|  | **SBP PRS only** | **Clinical only** | **PRS + clinical** | **C increase^#^** | **P-LRT*** |
| --- | --- | --- | --- | --- | --- |
| Hypertension | 0.622 (0.003) | 0.700 (0.003) | 0.717 (0.003) | 2.43% | < 0.00001 |
| Early-onset hypertension | 0.645 (0.008) | 0.838 (0.006) | 0.838 (0.006) | 0.00% | < 0.00001 |
| Late-onset hypertension | 0.607 (0.003) | 0.696 (0.003) | 0.707 (0.003) | 1.58% | < 0.00001 |
| Cardiovascular disease | 0.558 (0.003) | 0.590 (0.003) | 0.600 (0.003) | 1.69% | < 0.00001 |
| Myocardial infarction | 0.660 (0.005) | 0.710 (0.005) | 0.714 (0.005) | 0.56% | < 0.00001 |
| Stroke | 0.571 (0.006) | 0.610 (0.007) | 0.612 (0.006) | 0.33% | < 0.0001 |
| Chronic kidney disease | 0.608 (0.009) | 0.665 (0.009) | 0.667 (0.009) | 0.30% | < 0.01 |

*Results are presented as concordance index with standard error for each model. All models were adjusted for sex and the first 10 principal components, with age on the time scale. The systolic blood pressure (SBP) polygenic risk score (PRS) + clinical risk factors and clinical risk factors only models were also adjusted for total and high-density lipoprotein cholesterol, SBP, body mass index, use of antihypertensive medication, smoking, and non-fasting blood glucose. Data from the third wave of the Trøndelag Health Study (HUNT3; n = 49 228) were used. ^#^ Relative increase of concordance the concordance index when adding PRS data to the clinical models; ^*^ P-value for the likelihood ratio test (LRT) between the clinical only and PRS + clinical models. The LRT assesses model fit, with a lower p-value indicating better fit; the concordance index is a measure of its ability to discriminate between outcomes, with a higher C-value indicating greater discriminatory ability.*

**Table S15.** Model comparison with DBP PRS

|  | **DBP PRS only** | **Clinical only** | **PRS + clinical** | **C increase*** | **P-LRT*** |
| --- | --- | --- | --- | --- | --- |
| Hypertension | 0.612 (0.003) | 0.700 (0.003) | 0.714 (0.003) | 2.00% | < 0.00001 |
| Early-onset hypertension | 0.641 (0.008) | 0.838 (0.006) | 0.839 (0.006) | 0.12% | < 0.00001 |
| Late-onset hypertension | 0.598 (0.003) | 0.696 (0.003) | 0.705 (0.003) | 1.29% | < 0.00001 |
| Cardiovascular disease | 0.554 (0.003) | 0.590 (0.003) | 0.595 (0.003) | 0.85% | < 0.00001 |
| Myocardial infarction | 0.657 (0.005) | 0.710 (0.005) | 0.713 (0.005) | 0.42% | < 0.00001 |
| Stroke | 0.572 (0.006) | 0.610 (0.007) | 0.612 (0.006) | 0.33% | < 0.00001 |
| Chronic kidney disease | 0.602 (0.009) | 0.665 (0.009) | 0.666 (0.009) | 0.15% | 0.1607 |

*Results are presented as concordance index with standard error for each model. All models were adjusted for sex and the first 10 principal components, with age on the time scale. The diastolic blood pressure (DBP) polygenic risk score (PRS) + clinical risk factors and clinical risk factors only models were also adjusted for total and high-density lipoprotein cholesterol, SBP, body mass index, use of antihypertensive medication, smoking, and non-fasting blood glucose. Data from the third wave of the Trøndelag Health Study (HUNT3; n = 49 228) were used. ^#^ Relative increase of concordance the concordance index when adding PRS data to the clinical models; ^*^ P-value for the likelihood ratio test (LRT) between the clinical only and PRS + clinical models. The LRT assesses model fit, with a lower p-value indicating better fit; the concordance index is a measure of its ability to discriminate between outcomes, with a higher C-value indicating greater discriminatory ability.*

**Table S16.** Model comparison with both SBP and DBP PRS

|  | **Both PRS only** | **Clinical only** | **PRS + clinical** | **C increase*** | **P-LRT*** |
| --- | --- | --- | --- | --- | --- |
| Hypertension | 0.624 (0.003) | 0.700 (0.003) | 0.718 (0.003) | 2.57% | < 0.00001 |
| Early-onset hypertension | 0.652 (0.008) | 0.838 (0.006) | 0.839 (0.006) | 0.12% | < 0.00001 |
| Late-onset hypertension | 0.609 (0.003) | 0.696 (0.003) | 0.708 (0.003) | 1.72% | < 0.00001 |
| Cardiovascular disease | 0.558 (0.003) | 0.590 (0.003) | 0.600 (0.003) | 1.69% | < 0.00001 |
| Myocardial infarction | 0.660 (0.005) | 0.710 (0.005) | 0.714 (0.005) | 0.56% | < 0.00001 |
| Stroke | 0.573 (0.006) | 0.610 (0.007) | 0.612 (0.006) | 0.33% | < 0.00001 |
| Chronic kidney disease | 0.608 (0.009) | 0.665 (0.009) | 0.667 (0.009) | 0.30% | 0.0245 |

*Results are presented as concordance index with standard error for each model. All models were adjusted for sex and the first 10 principal components, with age on the time scale. The systolic blood pressure (SBP) and diastolic blood pressure (DBP) polygenic risk score (PRS) + clinical risk factors and clinical risk factors only models were also adjusted for total and high-density lipoprotein cholesterol, SBP, body mass index, use of antihypertensive medication, smoking, and non-fasting blood glucose. Data from the third wave of the Trøndelag Health Study (HUNT3; n = 49 228) were used. ^#^ Relative increase of concordance the concordance index when adding PRS data to the clinical models; ^*^ P-value for the likelihood ratio test (LRT) between the clinical only and PRS + clinical models. The LRT assesses model fit, with a lower p-value indicating better fit; the concordance index is a measure of its ability to discriminate between outcomes, with a higher C-value indicating greater discriminatory ability.*

# Results from repeat HUNT participants

**Table S17.** Descriptive data from repeat HUNT participants (n = 18 498; 56% female)

|  | **Age (y)** | **SBP (mmHg)** | **Medicated (%)** |
| --- | --- | --- | --- |
| **HUNT 1** |  |  |  |
| Low risk | 37.8 ± 9.4 | 120.6 ± 12.8 | 0.3 |
| Average risk | 37.2 ± 9.3 | 126.3 ± 14.8 | 1.5 |
| High risk | 36.9 ± 8.9 | 133.8 ± 17.6 | 4.9 |
| **HUNT 2** |  |  |  |
| Low risk | 49.2 ± 9.5 | 126.4 ± 16.0 | 2.1 |
| Average risk | 48.7 ± 9.3 | 134.2 ± 18.6 | 5.8 |
| High risk | 48.3 ± 8.9 | 144.7 ± 20.8 | 15.3 |
| **HUNT 3** |  |  |  |
| Low risk | 60.4 ± 9.4 | 127.5 ± 18.4 | 10.8 |
| Average risk | 59.8 ± 9.2 | 136.8 ± 20.1 | 21.6 |
| High risk | 59.5 ± 8.9 | 147.6 ± 20.8 | 39.6 |
| **HUNT 4** |  |  |  |
| Low risk | 71.1 ± 9.4 | 132.3 ± 20.6 | 21.4 |
| Average risk | 70.5 ± 9.2 | 142.1 ± 21.0 | 36.2 |
| High risk | 70.1 ± 8.8 | 151.4 ± 20.3 | 57.6 |

*Data presented as mean ± standard deviation. HUNT, the Trøndelag Health Study; SBP, systolic blood pressure; low risk = 1^st^ polygenic risk score (PRS) decile; average risk = 2^nd^ to 9^th^ PRS decile; high risk = 10^th^ PRS decile. The fraction of medicated individuals is calculated based on self-reported use of antihypertensive drugs at the time of the study.*

# Comparisons between HUNT and FinnGen

**Table S18.** Outcome comparison between HUNT4 and FinnGen

| **Outcome** | **HUNT4** | **FinnGen** |
| --- | --- | --- |
| Hypertension | 2.37 (2.11, 2.67) | 2.19 (2.10, 2.29) |
| Early-onset hypertension | 3.63 (2.93, 4.50) | 2.62 (2.48, 2.77) |
| Late-onset hypertension | 1.98 (1.72, 2.28) | 1.68 (1.57, 1.81) |
| Cardiovascular disease | 1.34 (1.19, 1.51) | 1.30 (1.22, 1.39) |
| Stroke | 1.36 (1.01, 1.84) | 1.29 (1.16, 1.44) |
| Chronic kidney disease | 1.43 (0.96, 2.14) | N/A |
| Myocardial infarction | 1.48 (1.13, 1.94) | N/A |

*HUNT, the Trøndelag Health Study. A comparison of hazard ratios (with 95% confidence intervals) between the top 2.5% of the polygenic risk score for systolic blood pressure (20-80% as reference) distribution between our study and Vaura et al.^12^*

**Table S19.** Sex-differences in HUNT4 and FinnGen

| **Outcome** | **Study population** | | | |
| --- | --- | --- | --- | --- |
|  | **HUNT4** | | **FinnGen** | |
| **Bottom 2.5% SBP PRS** | Women | Men | Women | Men |
| Hypertension | 0.32 (0.23, 0.45) | 0.49 (0.37, 0.65) | 0.41 (0.37, 0.45) | 0.61 (0.56, 0.67) |
| Early-onset hypertension | 0.23 (0.07, 0.72) | 0.28 (0.09, 0.86) | 0.33 (0.28, 0.40) | 0.60 (0.53, 0.69) |
| Late-onset hypertension | 0.34 (0.24, 0.48) | 0.53 (0.40, 0.71)* | 0.46 (0.40, 0.52) | 0.62 (0.55, 0.70) |
| **Top 2.5% SBP PRS** |  |  |  |  |
| Hypertension | 2.56 (2.19, 3.00) | 2.19 (1.84, 2.61) | 2.12 (1.99, 2.25) | 1.80 (1.69, 1.92) |
| Early-onset hypertension | 3.72 (2.77, 5.01) | 3.55 (2.60, 4.86) | 2.51 (2.32, 2.72) | 2.10 (1.94, 2.28) |
| Late-onset hypertension | 2.19 (1.81, 2.64) | 1.79 (1.45, 2.21) | 1.66 (1.50, 1.84) | 1.45 (1.30, 1.61) |

*HUNT, the Trøndelag Health Study; SBP, systolic blood pressure; PRS, polygenic risk score. A sex-specific comparison of hazard ratios (with 95% confidence intervals) between the top and bottom 2.5% of the PRS distribution for systolic blood pressure (20-80% as reference) distribution between our study and Kauko et al.^13^; ^*^ p < 0.05 for PRS × sex interaction.*

# References

1. Brumpton BM, Graham S, Surakka I*, et al.* The HUNT Study: a population-based cohort for genetic research. *medRxiv* 2021:2021.2012.2023.21268305. doi: 10.1101/2021.12.23.21268305

2. Das S, Forer L, Schönherr S*, et al.* Next-generation genotype imputation service and methods. *Nat Genet* 2016;**48**:1284-1287. doi: 10.1038/ng.3656

3. McCarthy S, Das S, Kretzschmar W*, et al.* A reference panel of 64,976 haplotypes for genotype imputation. *Nat Genet* 2016;**48**:1279-1283. doi: 10.1038/ng.3643

4. Kong A, Masson G, Frigge ML*, et al.* Detection of sharing by descent, long-range phasing and haplotype imputation. *Nature Genetics* 2008;**40**:1068-1075. doi: 10.1038/ng.216

5. Gudbjartsson DF, Helgason H, Gudjonsson SA*, et al.* Large-scale whole-genome sequencing of the Icelandic population. *Nature Genetics* 2015;**47**:435-444. doi: 10.1038/ng.3247

6. Vilhjálmsson Bjarni J, Yang J, Finucane Hilary K*, et al.* Modeling Linkage Disequilibrium Increases Accuracy of Polygenic Risk Scores. *The American Journal of Human Genetics* 2015;**97**:576-592. doi: <https://doi.org/10.1016/j.ajhg.2015.09.001>

7. Ge T, Chen C-Y, Ni Y, Feng Y-CA, Smoller JW. Polygenic prediction via Bayesian regression and continuous shrinkage priors. *Nature Communications* 2019;**10**:1776. doi: 10.1038/s41467-019-09718-5

8. Evangelou E, Warren HR, Mosen-Ansorena D*, et al.* Genetic analysis of over 1 million people identifies 535 new loci associated with blood pressure traits. *Nature Genetics* 2018;**50**:1412-1425. doi: 10.1038/s41588-018-0205-x

9. Gudmundsson H, Gudbjartsson DF, Frigge M, Gulcher JR, Stefánsson K. Inheritance of human longevity in Iceland. *Eur J Hum Genet* 2000;**8**:743-749. doi: 10.1038/sj.ejhg.5200527

10. Åsvold BO, Langhammer A, Rehn TA*, et al.* Cohort Profile Update: The HUNT Study, Norway. *medRxiv* 2021:2021.2010.2012.21264858. doi: 10.1101/2021.10.12.21264858

11. Fraser A, Macdonald-Wallis C, Tilling K*, et al.* Cohort Profile: the Avon Longitudinal Study of Parents and Children: ALSPAC mothers cohort. *Int J Epidemiol* 2013;**42**:97-110. doi: 10.1093/ije/dys066

12. Vaura F, Kauko A, Suvila K*, et al.* Polygenic Risk Scores Predict Hypertension Onset and Cardiovascular Risk. *Hypertension* 2021;**77**:1119-1127. doi: 10.1161/hypertensionaha.120.16471

13. Kauko A, Aittokallio J, Vaura F*, et al.* Sex Differences in Genetic Risk for Hypertension. *Hypertension* 2021;**78**:1153-1155. doi: 10.1161/hypertensionaha.121.17796
