# Supplementary material for: Polygenic risk scores associate with blood pressure traits across the lifespan
Source: Eur J Prev Cardiol. 2023 Nov 26;31(6):644–54. doi: 10.1093/eurjpc/zwad365 (PMC11025038; doi:10.1093/eurjpc/zwad365)
Supplement: zwad365_Supplementary_Data [file zwad365_supplementary_data.zip › Translational perspective - R2.docx]

**Translational perspective**

This study developed and compared multiple polygenic risk scores for blood pressure traits. After identifying the best performing score for each trait, they were applied in downstream analysis in large cohorts of adults and children. The findings showed that polygenic risk scores may aid primordial prevention by identifying individuals more likely to develop high blood pressure early in life with a consequent increase in the risk for hypertension-related morbidity and mortality later in life. Combining genetic and phenotypic risk profiles also indicated that the increased risk is driven by phenotypic expression and not genetic risk per se, highlighting the importance of blood pressure management throughout life.
